# Incorporation of next-generation sequencing in clinical practice using solid and liquid biopsy for patients with non-Hodgkin’s lymphoma

**DOI:** 10.1038/s41598-021-02362-4

**Published:** 2021-11-24

**Authors:** Mariana Bastos-Oreiro, Julia Suárez-González, Cristina Andrés-Zayas, Natalia Carolina Carrión, Solsiré Moreno, Diego Carbonell, María Chicano, Paula Muñiz, Laura Sanz, Francisco Javier Diaz-Crespo, Javier Menarguez, José Luis Diez-Martín, Ismael Buño, Carolina Martínez-Laperche

**Affiliations:** 1 Department of Hematology, Gregorio Marañón General University Hospital, C/ Doctor Esquerdo 46, 28007 Madrid, Spain; 2 Gregorio Marañón Health Research Institute (IiSGM), Madrid, Spain; 3 Genomics Unit, Gregorio Marañón General University Hospital, Gregorio Marañón Health Research Institute (IiSGM), Madrid, Spain; 4 Deparment of Pathology, Gregorio Marañón General University Hospital, Madrid, Spain; 5 Department of Medicine, School of Medicine, Complutense University of Madrid, Madrid, Spain; 6 Department of Cell Biology, School of Medicine, Complutense University of Madrid, Madrid, Spain

**Keywords:** B-cell lymphoma, Cancer genetics

## Abstract

Although next-generation sequencing (NGS) data on lymphomas require further validation before being implemented in daily practice, the clinical application of NGS can be considered right around the corner. The aim of our study was to validate an NGS lymphoid panel for tissue and liquid biopsy with the most common types of non-Hodgkin’s lymphoma [follicular lymphoma (FL) and diffuse large B-cell lymphoma (DLBCL)]. In this series, 372 somatic alterations were detected in 93.6% (44/47) of the patients through tissue biopsy. In FL, we identified 93 somatic alterations, with a median of 7.4 mutations per sample. In DLBCL, we detected 279 somatic variants with a median of 8.6 mutations (range 0–35). In 92% (24/26) of the cases, we were able to detect some variant in the circulating tumor DNA. We detected a total of 386 variants; 63.7% were detected in both types of samples, 13.2% were detected only in the circulating tumor DNA, and 23% were detected only in the tissue biopsy. We found a correlation between the number of circulating tumor DNA mutations, advanced stage, and bulky disease. The genetic alterations detected in this panel were consistent with those previously described at diagnosis. The liquid biopsy sample is therefore a complementary tool that can provide new genetic information, even in cases where a solid biopsy cannot be performed or an insufficient sample was obtained. In summary, we describe and analyze in this study the findings and difficulties encountered when incorporating liquid biopsy into clinical practice in non-Hodgkin’s lymphoma at diagnosis.

## Introduction

Lymphoproliferative disorders are a large and heterogeneous group of hematological malignancies. Mature B-cell lymphoproliferative syndromes comprise 80% of all lymphomas^1^. Diffuse large B-cell lymphoma (DLBCL) and follicular lymphoma (FL) are the two most common types of lymphoma^1^. The diagnosis, prognosis assessment and efficacy evaluation mainly depend on tissue biopsy, laboratory data, and imaging tests. Classically, the biopsy assessment includes the immunohistochemical detection of markers and fluorescence in situ hybridization studies (*MYC*, *BCL6*, and *BCL2* rearrangements)^2–6^. Continuous efforts are being made to identify genomic biomarkers to better understand the behavior of lymphomas, predict their evolution and bring solutions to clinical practice. Next-generation sequencing (NGS), which allows for massive, parallel, high-throughput DNA sequencing, has emerged over the past decade and has provided new insights into the genomic and transcriptomic characterization of mature B-cell malignancies^8^. NGS has become a useful tool for the complete characterization of the spectrum of genetic variants in non-Hodgkin’s lymphoma (NHL). Research on molecular profiles in NHL has advanced significantly in recent years. Various groups have attempted to establish prognostic scores^3^ and genetic risk clusters based on genetic characteristics^4^ or by combining the characteristics with clinical and analytical data^5,6^. The results of these studies are promising; however, the means to apply these technologies are still limited in most centers, and validation is required for implementation into clinical practice. Thus, while NGS lymphoid panels should be implemented in clinical practice, there is as yet no standard approach, and features such as gene selection, sequencing platform, read depth, and variant analysis can differ among laboratories.

Although tissue biopsy is the gold standard for identifying genetic variants, it might not reflect the entire molecular complexity of every patient with lymphoma^7,8^. Once the diagnosis of lymphoma is reached based on a tissue biopsy, a liquid biopsy can be applied to complement the tissue findings. Liquid biopsy, which is non-invasive, can also be used to explore the entire mutational landscape of the lymphoma, given that this approach has the potential for collecting the tumor-circulating tumor DNA (ctDNA) derived from most, and potentially all, tumor locations in the body. Liquid biopsy has thereby progressively transformed cancer diagnoses and prognoses, as well as oncologic therapy in general and lymphoma in particular^9^. This technique is expected to lead to important improvements in initial risk stratification, response evaluation at the end of induction therapy, and in surveillance strategies and target therapy selection in patients with lymphoma.

The aim of our study was to validate an NGS lymphoid panel for solid and liquid biopsy in the most common NHLs (DLBCL and FL) and to assess the concordance between genetic mutations detected in solid and liquid biopsies.

## Patients and methods

### Clinical cohort

The study included 47 nonconsecutive patients diagnosed with NHL 32 DLBCL [20 DLBCLs-not otherwise specified (NOS), 4 high-grade double-hit lymphomas, 5 high-grade NOS lymphomas and 3 primary mediastinal B-cell lymphomas] and 15 FL from 2014 to 2019 in Gregorio Marañón General University Hospital. The study also included formalin-fixed paraffin-embedded (FFPE) tissue samples from the time of diagnosis, along with matched same-day plasma samples from 26 of these patients (14 DLBCL-NOS, 4 high grade) (Supplementary Tables 1 and 2).

Ethics approval was obtained from the Research Ethics Committee of Gregorio Marañón General University Hospital. All patients provided written informed consent according to the principles of the Declaration of Helsinki.

### DNA extraction

All FFPE sections (n = 47) were subjected to DNA extraction with the QIAGEN Generead DNA FFPE Kit (QIAGEN, Germany) according to the manufacturer’s guidelines. Peripheral blood samples were collected from 26 patients, placed in 10 mL EDTA tubes and centrifuged at 1800 × g for 10 min to isolate plasma, which was aliquoted into 1.5–2-mL tubes and stored at − 80 °C. Cell free DNA (ctDNA) was extracted with a QIAamp circulating nucleic acid kit (QIAGEN, Germany). FFPE and ctDNA were quantified using a Qubit dsDNA BR Assay (THERMO FISHER SCIENTIFIC, Waltham, MA, USA).

### NGS experiments and data analysis

We selected The Lymphoma Solution (SOPHIA GENETICS, Switzerland) targeted panel, given that it targets 54 relevant genes in lymphomagenesis (193 kb) (Supplementary Table 3). For each FFPE tissue sample, 32–100 ng of total DNA was used to prepare the library according to the manufacturer’s protocol. Pools of up to 12 purified libraries were captured. For each circulating tumour DNA (ctDNA) sample, 2.5–55 ng of circulating DNA was used to prepare the library. Due to the intrinsic characteristics of the ctDNA samples, adapter ligation was performed directly without initial DNA fragmentation, followed by hybridization with the capture probes, also in pools of up to 12 purified libraries. Lastly, two capture pools (24 samples) were sequenced on a NextSeq platform (ILLUMINA, US; Paired-end 2 × 151 bp; mid-output kit).

We used the Sophia DDM platform (SOPHIA GENETICS, Switzerland) to analyze single nucleotide variants and small insertions and deletions. FASTQ files were uploaded to the data portal and aligned with the human reference genome (GRCh37/hg19). After annotation in DDM, non-synonymous variants located in exonic or ± 1.2 intronic splice regions were retained, and variants with a minor allele frequency < 0.01 (based on ExAC, GnomAD and 1000 Genomes databases), were selected for the downstream analysis. Currently, there is no standardization to establish which is the best cut-off point for VAF. In this sense, we decided to set the percentage at 5% in the FFPE since there are a high percentage of tumor in these samples, in an attempt to avoid false positives. However, the cut-off was reduced to 1% in the plasma samples where there is a lower percentage of tumor and we could lose mutations.

We used an Integrative Genomics Viewer (Broad Institute, USA) to visualize the variants aligned against the reference genome to confirm the accuracy of the variant calls by checking for possible strand biases and sequencing errors. Copy number variations (CNVs) were not analyzed in this study.

The ctDNA concentrations were expressed in haploid genome equivalents (hGE) per mL of plasma (hGE/mL) and were calculated by multiplying the mean VAF for all mutations used for detection calling by the concentration of cfDNA (pg/mL of plasma) and dividing by 3.3, using the assumption that each haploid genomic equivalent weighed 3.3 pg, as previously described by Scherer et al. (Supplementary Table 5).

### Test validation

For technical validation, input DNA requirements, library generation and sequencing, two rounds of validation were performed consecutively. Three previously characterized samples with known single nucleotide variants and/or indels, as in the 24 FFPE tissue samples, were analyzed. Multiple intercapture and intracapture replicates, as well as inter-run and intra-run replicates were included (data not shown).

### Statistical analysis

The patient characteristics are presented as frequencies (n) and percentages (%) for categorical variables or as medians and ranges for continuous variables. Categorical data were compared with Fisher’s exact or chi-squared test, when appropriate, and continuous data were compared using a two-tailed paired Mann Whitney U test. R Statistical Software was used for all statistical tests. Probability values < 0.05 were considered significant.

## Results

### Gene panel features

A total of 73 samples (47 FFPE and 26 ctDNA) were sequenced, resulting in a median of 8,290,518 reads in the FFPE samples and 11,071,271 in the ctDNA samples. The median percentage of mapped reads was 97% in both types of samples (Supplementary Table 4). The median percentage of mapped base pairs on-target was 83% in the FFPE samples and 73% in the ctDNA samples. The median percentage of duplicate fragments per sample was 35% in the FFPE samples and 62% in the ctDNA samples. The median deep coverage of target regions was 2101x (range 231x–6518x) in the FFPE samples (median coverage heterogeneity of 0.04%) and 3678x (range 1906x–9270x) in the ctDNA samples (median coverage heterogeneity of 0.24%) (Supplementary Table 4).

### Mutational data from the FFPE samples (n = 47)

**Figure 1:**
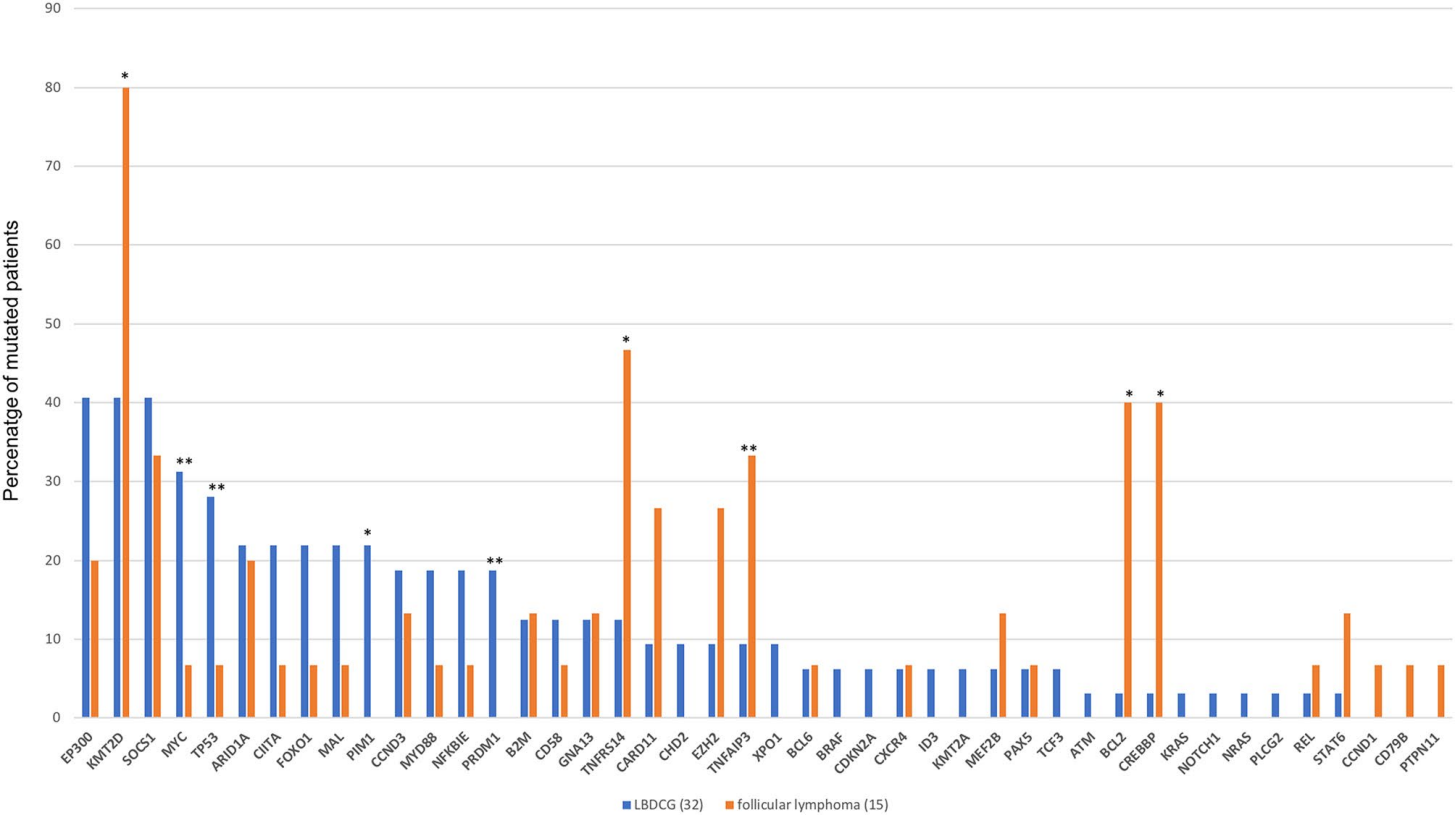
Frequencies of mutated genes in the cohort (n = 47). Significant differences between follicular lymphoma and diffuse large B-cell lymphoma (*p* < 0.05*) (*p* < 0.1**).

The gene panel was performed on 47 patients with NHL; 93.6% (44/47) presented at least one variant in the FFPE tissue samples with VAF ≥ 5%. In total, 372 somatic alterations were detected (Table 1). The patients presented a median of 6 mutations per sample (range 0–37). Missense mutations were the most frequent at 253/372 (67.6%), followed by 48/372 (12.9%) frameshift mutations, and 34/372 (9.1%) nonsense mutations (Table 1). Figure 1 and Supplementary Figs. 1 and 2 present the gene frequencies by NHL subtype detected in the total cohort.

**Table 1:** Mutational analysis of formalin-fixed paraffin-embedded tissue samples.

UPN	Type of sample	Diagnosis	Gene	c.DNA	Protein	VAF	Consenquence
1	FFPE	FL	*B2M*	c.20T > G	p.(Leu7*)	62.8	Nonsense
			*CREBBP*	c.4445A > G	p.(Lys1482Arg)	30.2	Missense
			*PAX5*	c.491_577del	p.(Val164_Ala192del)	19.6	Inframe
			*REL*	c.1265_1273dupATTTAAATG	p.(Asp422_Ans424dup)	13.2	Inframe
			*SOCS1*	c.3_13delGGTAGCACACA	p.(Met?)	28.4	No-start
			*SOCS1*	c.180_181delGC	p.(His61Argfs*55)	30.3	Frameshift
2	FFPE	DLBCL	*CARD11*	c.746A > C	p.(Gln249Pro)	43	Missense
			*CREBBP*	c.4393T > G	p.(Tyr1465Asp)	89.4	Missense
			*EZH2*	c.1921T > C	p.(Tyr641His)	42.1	Missense
			*TNFRSF14*	c.602G > A	p.(Trp201*)	79.1	Nonsense
			*ID3*	c.256G > C	p.(Glu86Gln)	46.4	Missense
			*KMT2D*	c.8607_8608insAAGGC	p.(Gly2870Lysfs*42)	27.7	Frameshift
3	FFPE	FL	*TP53*	c.743G > T	p.(Arg248Leu)	35.1	Missense
			*CD79B*	c.600delC	p.(Asp200Glufs*11)	72.6	Frameshift
			*NFKBIE*	c.1094delT	p.(Leu365Argfs*66)	13.4	Frameshift
			*EP300*	c.3014G > A	p.(Cys1005Tyr)	43.1	Missense
			*KMT2D*	c.4569C > A	p.(Cys1523*)	29.3	Nonsense
4	FFPE	FL	*BCL2*	c.140G > A	p.(Gly47Asp)	28	Missense
			*BCL2*	c.175C > A	p.(Pro59Thr)	28.4	Missense
			*BCL2*	c.151T > G	p.(Ser51Ala)	28.8	Missense
			*EZH2*	c.1922A > T	p.(Tyr641Phe)	30.5	Missense
			*STAT6*	c.1256A > G	p.(Asp419Gly)	5.3	Missense
			*CD58*	c.254C > G	p.(Thr85Ser)	47.9	Missense
			*MEF2B*	c.170A > G	p.(Tyr57Cys)	16.8	Missense
			*KMT2D*	c.3931A > T	p.(Arg1311*)	42.2	Nonsense
			*KMT2D*	c.13893 + 2T > A	p.(?)	22.4	Splice_donor_ + 2
5	FFPE	DLBCL	*TP53*	c.715A > G	p.(Asn239Asp)	5.9	Missense
			*TP53*	c.536A > C	p.(His179Pro)	7.9	Missense
			*NOTCH2*	c.4568A > G	p.(Asn1523Ser)	46.8	Missense
			*TCF3*	c.1634G > A	p.(Arg545Gln)	49.1	Missense
			*TCF3*	c.1670T > A	p.(Val557Glu)	6.2	Missense
			*MYC*	c.55A > C	p.(Val19Leu)	7.8	Missense
6	FFPE	DLBCL	–	–	–	–	–
7	FFPE	DLBCL	*BCL6*	c.1753C > T	p.(Arg585Trp)	12.2	Missense
			*BCL6*	c.1853G > A	p.(Arg618His)	11.9	Missense
			*CREBBP*	c.4925_4927del	p.(Ser1642del)	15	Inframe
			*CCND3*	c.568dupC	p.(Arg190Profs*)	36.4	Frameshift
			*CD58*	c.493C > T	p.(Gln165*)	53.8	Nonsense
			*CDKN2A*	c.172C > T	p.(Arg58*)	52.2	Nonsense
			*CIITA*	c.1801C > T	p.(Arg601Trp)	32.3	Missense
			*EP300*	c.4879C > T	p.(Arg1627Trp)	12.3	Missense
			*GNA13*	c.220C > T	p.(Gln74*)	71.8	Nonsense
8	FFPE	FL	*SOCS1*	c.299C > T	p.(Thr100Ile)	11.8	Missense
			*MAL*	c.271T > C	p.(Tyr91His)	61.7	Missense
			*KMT2D*	c.3535_3539delGGCTinsAACCATGTGAAGA	p.(Gly1179AsnFs*36)	13	Frameshift
9	FFPE	DLBCL	*CDKN2A*	c.329G > A	p.(Trp110*)	6.9	Nonsense
			*EP300*	c.631G > A	p.(Gly211Ser)	43.3	Missense
			*KMT2D*	c.14450T > G	p.(Val4817Gly)	51.3	Missense
10	FFPE	DLBCL	*CARD11*	c.1070A > T	p.(Asp357Val)	12.9	Missense
			*XPO1*	c.1711G > A	p.(Glu571Lys)	13	Missense
			*TNFRSF14*	c.608G > A	p.(Trp203*)	12.4	Nonsense
			*CCND3*	c.605C > T	p.(Thr202Ile)	11.3	Missense
			*CHD2*	c.1397G > A	p.(Arg466Gln)	12.7	Missense
			*EP300*	c.4454A > T	p.(Asp1485Val)	8.7	Missense
11	FFPE	FL	*BCL2*	c.392C > G	p.(Ala131Gly)	11.7	Missense
			*BCL2*	c.517A > G	p.(Ile173Val)	9.4	Missense
			*CARD11*	c.748T > C	p.(Ser250Pro)	14.5	Missense
			*CREBBP*	c.4394A > G	p.(Tyr1465Cys)	32.1	Missense
			*CCND3*	c.531_532delCTinsTG	p.(Ser178Ala)	99.7	Missense
			*KMT2D*	c.6664C > T	p.(Gln2222*)	12.2	Nonsense
			*KMT2D*	c.5335A > T	p.(Lys1779*)	14.5	Nonsense
12	FFPE	FL	*CARD11*	c.864 + 1G > C	p.(?)	34.8	Splice_donor_ + 1
			*CREBBP*	c.4336T > G	p.(Phe1446Val)	36.7	Missense
			*STAT6*	c.1256A > C	p.(Asp419Ala)	16.2	Missense
			*TNFAIP3*	c.1706G > A	p.(Arg569Gln)	33.3	Missense
			*CIITA*	c.2342_2345delCGGTinsTGGC	p.(Ser781_Val782delinsLeuAla)	20.7	Missense
			*KMT2D*	c.11456_11474del	p.(Gly3819AspFs*15)	13	Frameshift
			*KMT2D*	c.9781C > T	p.(Gln3261*)	13.6	Nonsense
13	FFPE	DLBCL	*TP53*	c.490A > T	p.(Lys164*)	31.8	Nonsense
			*B2M*	c.2T > G	p.(Met1?)	52.4	No-start
			*PIM1*	c.676G > A	p.(Glu226Lys)	35.5	Missense
			*PIM1*	c.370C > T	p.(Pro124Ser)	62.1	Missense
			*PIM1*	c.434G > A	p.(Arg145His)	6.7	Missense
			*PIM1*	c.202C > T	p.(His68Tyr)	23.3	Missense
			*SOCS1*	c.8C > T	p.(Ala3Val)	30.3	Missense
			*FOXO1*	c.435del	p.(Ala146Argfs*187)	17.9	Frameshift
			*MEF2B*	c.78C > G	p.(Phe26Leu)	52.5	Missense
			*MYD88*	c.818T > C	p.(Leu273Pro)	33	Missense
14	FFPE	FL	*BCL2*	c.191A > C	p.(Asp64Ala)	12	Missense
			*BCL2*	c.93T > C	p.(Asp31Glu)	9	Missense
			*TNFAIP3*	c.2014G > T	p.(Gly672*)	8.8	Nonsense
			*TNFRSF14*	c.463delA	p.(Thr155Profs*)	13.3	Frameshift
			*KMT2D*	c.172 + 2T > C	p.(?)	12.4	Splice_donor_ + 2
15	FFPE	FL	*CREBBP*	c.4382T > C	p.(Leu1461Pro)	29.1	Missense
			*SOCS1*	c.630G > C	p.(Gln210His)	47.6	Missense
			*KMT2D*	c.16489_16491delATC	p.(Ile5479del)	32.4	Inframe
			*KMT2D*	c.9019delG	p.(Glu3007Lysfs*22)	26.1	Frameshift
16	FFPE	FL	*CCND1*	c.31G > T	p.(Glu11*)	44.9	Nonsense
			*TNFRSF14*	c.29-1G > A	p.(?)	40.7	Splice_acceptor_-1
			*KMT2D*	c.15143G > A	p.(Arg5048His)	27	Missense
			*KMT2D*	c.8401C > T	p.(Arg2801*)	26.4	Nonsense
			*PRDM1*	c.351A > G	p.(Ile117Met)	46.7	Missense
17	FFPE	FL	*BCL2*	c.19_21delinsGCG	p.(Thr7Ala)	24.7	Missense
			*CARD11*	c.752T > C	p.(Leu251Pro)	32.7	Missense
			*CREBBP*	c.4925_4927del	p.(Ser1642del)	21.1	Inframe
			*EZH2*	c.1921T > A	p.(Tyr641Asn)	36.4	Missense
			*PTPN11*	c.1165A > C	p.(Lys389Gln)	7.6	Missense
			*ARID1A*	c.60_62del	p.(Pro21del)	7.3	Inframe
18	FFPE	DLBCL	*NFKBIE*	c.668_671delTGCTinsAGCG	p.(Leu223_Leu224delins*Arg)	20.6	Missense
			*SOCS1*	c.7G > A	p.(Ala3Thr)	14	Missense
			*SOCS1*	c.374G > C	p.(Ser125Thr)	19.1	Missense
			*SOCS1*	c.407A > C	p.(His136Pro)	18	Missense
			*SOCS1*	c.428G > A	p.(Ser143Asn)	13.5	Missense
			*SOCS1*	c.398delG	p.(Gly133Alafs*72)	10.6	Frameshift
			*SOCS1*	c.55C > T	p.(Pro19Ser)	14.6	Missense
			*SOCS1*	c.412G > C	p.(Asp138His)	15.9	Missense
			*SOCS1*	c.391C > G	p.(Gln131Glu)	16.4	Missense
			*SOCS1*	c.391C > T	p.(Gln131*)	16.4	Nonsense
			*SOCS1*	c.318C > G	p.(Ser106Arg)	8.7	Missense
			*SOCS1*	c.528G > C	p.(Glu176Asp)	10.6	Missense
			*ARID1A*	c.5012G > A	p.(Arg1671Gln)	18.4	Missense
			*EP300*	c.2359G > A	p.(Gly787Ser)	44.2	Missense
			*MYC*	c.482C > T	p.(Ser161Leu)	10.9	Missense
			*MYC*	c.218_219delCCinsTA	p.(Thr73Ile)	11.2	Missense
			*MYC*	c.1164C > G	p.(Ser388Arg)	18.7	Missense
			*MYC*	c.557G > C	p.(Cys186Ser)	14.5	Missense
			*MYC*	c.895G > C	p.(Ala299Pro)	18.6	Missense
			*MYC*	c.910_999dup	p.(Lys304_Asp333dup)	40.6	Inframe
			*MYC*	c.654C > G	p.(Ser218Arg)	15.1	Missense
			*MYC*	c.785C > T	p.(Thr262Ile)	15.8	Missense
			*MYC*	c.68_71delinsGCAG	p.(Phe23Cys)	9.7	Missense
			*MYC*	c.63C > G	p.(Ser21Arg)	9.6	Missense
			*MYC*	c.162G > C	p.(Glu54Asp)	9	Missense
			*MYC*	c.144G > A	p.(Asp48Glu)	7.9	Missense
			*MYC*	c.358_361delinsTTGT	p.(Asp120Leu)	11.8	Missense
19	FFPE	FL	*CARD11*	c.1202A > T	p.(Asp401Val)	19.9	Missense
			*SOCS1*	c.4G > T	p.(Val2Leu)	18.1	Missense
			*SOCS1*	c.14A > G	p.(Asn5Ser)	20	Missense
			*SOCS1*	c.134_139dupTCCCGG	p.(Val45_Pro46dup)	43.5	Inframe
			*TNFAIP3*	c.1035C > A	p.(Tyr345*)	28.9	Nonsense
			*TNFRSF14*	c.70G > T	p.(Val24Leu)	28.1	Missense
20	FFPE	DLBCL	*XPO1*	c.1711G > A	p.(Glu571Lys)	7	Missense
			*NFKBIE*	c.759_762delTTAC	p.(Tyr254Serfs*13)	17.9	Frameshift
			*PAX5*	c.548G > C	p.(Gly183Ala)	7.6	Missense
			*PIM1*	c.3G > A	p.(Met1?)	7.3	No-start
			*PIM1*	c.111G > T	p.(Gln37His)	7.6	Missense
			*PIM1*	c.224C > T	p.(Ser75Phe)	7.3	Missense
			*PIM1*	c.290G > C	p.(Ser97Thr)	9.8	Missense
			*PIM1*	c.379C > A	p.(Gln127Lys)	6.3	Missense
			*PIM1*	c.73C > G	p.(Leu25Val)	6.3	Missense
			*SOCS1*	c.195_197delinsACC	p.(Arg66Pro)	5.5	Missense
			*SOCS1*	c.275G > C	p.(Arg92Pro)	5.1	Missense
			*SOCS1*	c.17A > C	p.(Gln6Pro)	5.3	Missense
			*SOCS1*	c.46_49delinsTCAA	p.(Ala16_Ala17delinsSerThr)	7	Missense
			*SOCS1*	c.387C > G	p.(His129Gln)	5.7	Missense
			*SOCS1*	c.416G > C	p.(Gly139Ala)	6.9	Missense
			*SOCS1*	c.356T > C	p.(Met119Thr)	5.8	Missense
			*ARID1A*	c.3999_4001del	p.(Gln1334del)	7.5	Inframe
			*CIITA*	c.52 + 1G > T	p.(?)	10.7	Splice_donor_ + 1
			*CIITA*	c.2342_2345delCGGTinsTGGC	p.(Ser781_Val782delinsLeuAla)	35.8	Missense
			*CIITA*	c.3127_3134del	p.(Ala1043Profs*9)	8.4	Frameshift
			*MYC*	c.680A > C	p.(Asp227Ala)	10	Missense
21	FFPE	DLBCL	*B2M*	c.176T > A	p.(Leu59*)	80.3	Nonsense
			*ATM*	c.8284C > T	p.(Gln2762*)	54.3	Nonsense
			*NFKBIE*	c.1147_1153delCAACCAC	p.(Gln383Serfs*46)	31.3	Frameshift
			*NFKBIE*	c.759_762delTTAC	p.(Tyr254Serfs*13)	33.4	Frameshift
			*PRDM1*	c.75delG	p.(Arg25Serfs*13)	41.8	Frameshift
			*SOCS1*	c.358_361delGCCTinsCC	p.(Ala120Profs*?)	39.7	Frameshift
			*SOCS1*	c.434_437delACTG	p.(Asp145Alafs*59)	39.4	Frameshift
			*TNFAIP3*	c.2350C > T	p.(Gln784*)	64	Nonsense
			*TNFAIP3*	C.295 + 2T > C	p.(?)	67.5	Splice_donor_ + 2
			*CDKN2A*	c.394G > A	p.(Ala132Thr)	32.4	Missense
			*CIITA*	c.34_46delTACCTGTCAGAGC	p.(Tyr12Profs*15)	36.4	Frameshift
			*CIITA*	c.1652delG	p.(Gly551Alafs*7)	35.9	Frameshift
			*CIITA*	c.3262G > A	p.(Gly1880Arg)	5.3	Missense
			*FOXO1*	c.61C > T	p.(Arg21Cys)	39.4	Missense
			*GNA13*	c.179A > G	p.(Asp60Gly)	81.6	Missense
			*MYC*	c.25A > G	p.(Asn9Asp)	43.3	Missense
22	FFPE	FL	*BCL6*	c.1752C > A	p.(Asn584Lys)	27.8	Missense
			*CCND3*	c.613G > A	p.(Asp205Asn)	5.3	Missense
			*GNA13*	c.243_244del	p.(Glu82Glyfs*19)	5	Frameshift
			*MYC*	c.154_156del	p.(Lys51Del)	5.4	Inframe
23	FFPE	DLBCL	*TP53*	c.839G > A	p.(Arg280Lys)	28.2	Missense
			*NFKBIE*	c.759_762delTTAC	p.(Tyr254Serfs*13)	12.3	Frameshift
			*CCND3*	c.626T > C	p.(Ile209Thr)	23.1	Missense
			*CCND3*	c.604A > C	p.(Thr202Pro)	24.4	Missense
			*KMT2D*	c.10919G > A	p.(Gly3640Glu)	58.5	Missense
24	FFPE	DLBCL	*BCL2*	c.17G > A	p.(Arg6Lys)	5.9	Missense
			*B2M*	c.16G > C	p.(Ala6Pro)	49.4	Missense
			*TNFRSF14*	c.49_50delinsCAG	p.(Lys17Glnfs*60)	8.9	Frameshift
			*CXCR4*	c.1025C > A	p.(Ser342*)	5	Nonsense
25	FFPE	DLBCL	*TP53*	c.743G > A	p.(Arg248Gln)	66.3	Missense
			*SOCS1*	c.8C > T	p.(Ala3Val)	38.8	Missense
			*EP300*	c.631G > A	p.(Gly211Ser)	49.5	Missense
			*KMT2D*	c.13139delC	p.(Pro4380Glnfs*4)	42.3	Frameshift
			*MYD88*	c.818T > C	p.(Leu273Pro)	76.3	Missense
26	FFPE	DLBCL	*TP53*	c.725G > T	p.(Cys242Phe)	76.8	Missense
			*TCF3*	c.1688G > A	p.(Arg563His)	17.1	Missense
27	FFPE	DLBCL	*XPO1*	c.1711G > C	p.(Glu571Lys)	8.6	Missense
			*KMT2D*	c.7547C > G	p.(Pro2516Arg)	45.2	Missense
			*KMT2D*	c.7604G > A	p.(Arg2535His)	39.2	Missense
28	FFPE	DLBCL	*CHD2*	c.1281G > A	p.(Trp427*)	18.6	Nonsense
			*MAL*	c.98T > G	p.(Phe33Cys)	6	Missense
			*MYC*	c.1148A > G	p.(Asn383Ser)	22.9	Missense
			*MYC*	c.490C > T	p.(Pro164Ser)	20.6	Missense
			*MYD88*	c.818T > C	p.(Leu273Pro)	18.4	Missense
			*MYD88*	c.797C > T	p.(Pro266Leu)	19.1	Missense
29	FFPE	DLBCL	*PIM1*	c.403G > T	p.(Glu135*)	36.6	Nonsense
			*PIM1*	c.3G > T	p.(Met?)	34.8	No-start
			*PIM1*	c.544C > G	p.(Leu182Val)	33.7	Missense
			*PIM1*	c.111G > C	p.(Gln37His)	34.7	Missense
			*PIM1*	c.550C > G	p.(Leu184Val)	33.8	Missense
			*PIM1*	c.455T > A	p.(Leu152Gln)	38	Missense
			*PIM1*	c.382G > A	p.(Asp128Asn)	36.4	Missense
			*PIM1*	c.300C > G	p.(Phe100Leu)	35.7	Missense
			*PIM1*	c.83G > T	p.(Gly28Val)	30.5	Missense
			*PIM1*	c.424_427delGAGCinsCAGG	p.(Glu142_Leu143delinsGlnVal)	31.3	Missense
			*SOCS1*	c.564_565delCG	p.(Asn190Profs*?)	25.4	Frameshift
			*SOCS1*	c.213_220delCGCGCTCCinsT	p.(Ala72Trpfs*11)	44.8	Frameshift
			*SOCS1*	c.523C > T	p.(Gln175*)	23.5	Nonsense
			*SOCS1*	c.264_437del	p.(Ala89_Cys146del)	23	Inframe
			*SOCS1*	c.18G > C	p.(Gln6His)	44.9	Missense
			*SOCS1*	c.318C > A	p.(Ser106Arg)	30.4	Missense
			*SOCS1*	c.4_7delGTAGinsCTAC	p.(Val2_Ala3delinsLeuPro)	22.8	Missense
			*SOCS1*	c.195_197delGCGinsACA	p.(Arg66His)	23	Missense
			*SOCS1*	c.237C > G	p.(Phe79Leu)	42.9	Missense
			*SOCS1*	c.255C > G	p.(Ser85Arg)	38.2	Missense
			*SOCS1*	c.529C > G	p.(Leu177Val)	23.9	Missense
			*SOCS1*	c.254G > C	p.(Ser85Thr)	28.4	Missense
			*SOCS1*	c.176G > C	p.(Arg59Pro)	23.2	Missense
			*SOCS1*	c.391C > G	p.(Gln131Glu)	28.2	Missense
			*SOCS1*	c.4_6delGTAinsTTG	p.(Val2Leu)	43.3	Missense
			*CD58*	c.23_24dup	p.(Arg9Glyfs*34)	7.1	Frameshift
			*CHD2*	c.3976G > A	p.(Glu1326Lys)	43	Missense
			*MYC*	c.214C > T	p.(Pro72Ser)	34.5	Missense
			*MYC*	c.490C > G	p.(Leu164Val)	49.6	Missense
			*MYC*	c.245_246delCCinsTA	p.(Ser82Leu)	45.6	Missense
			*MYC*	c.223C > G	p.(Pro75Ala)	35.3	Missense
			*MYC*	c.963G > C	p.(Gln321His)	47.5	Missense
			*MYC*	c.569G > C	p.(Ser190Thr)	50.4	Missense
			*MYC*	c.358C > G	p.(Leu120Val)	47.5	Missense
			*MYC*	c.763C > T	p.(Leu255Phe)	48.4	Missense
			*MYC*	c.221_223delinsAGG	p.(Tyr74*)	8.3	Nonsense
			*MYC*	c.474G > A	p.(Asp158Glu)	48.3	Missense
30	FFPE	DLBCL	*MAL*	c.98T > C	p.(Phe33Cys)	6.9	Missense
31	FFPE	DLBCL	*BRAF*	c.1780G > A	p.(Asp594Asn)	29.4	Missense
			*EZH2*	c.1921T > A	p.(Tyr641Asn)	34.4	Missense
			*STAT6*	c.1255G > T	p.(Asp419Tyr)	52.4	Missense
			*PIM1*	c.409G > T	p.(Gly137*)	34	Nonsense
			*PIM1*	c.285G > C	p.(Lys95Asn)	31.8	Missense
			*SOCS1*	c.220C > G	p.(Leu74Val)	30.7	Missense
			*SOCS1*	c.178T > C	p.(Ser60Pro)	28.6	Missense
			*CIITA*	c.2342_2345delCGGTinsTGGC	p.(Ser781_Val782delinsLeuAla)	43.7	Missense
			*EP300*	c.6091C > T	p.(Pro2031Ser)	48.4	Missense
			*KMT2D*	c.14843C > G	p.(Ser4948*)	40.3	Nonsense
			*KMT2D*	c.7586delG	p.(Gly2529Alafs*14)	22.7	Frameshift
32	FFPE	DLBCL	*NOTCH1*	c.7541_7542delCT	p.(Pro2514Argfs*4)	34.7	Frameshift
			*ARID1A*	c.4540_4543delACGGinsCCGT	p.(Thr1514_Gly1515delinsProCys)	10.1	Missense
			*MAL*	c.98T > C	p.(Phe33Cys)	6	Missense
			*KMT2D*	c.2886_2887delTGinsCA	p.(Ala963Thr)	11.5	Missense
33	FFPE	DLBCL	*NFKBIE*	c.98C > T	p.(Ser33Phe)	63	Missense
			*CCND3*	c.544_554dupTCCAGCCCAGC	p.(Lys187Alafs*?)	63.7	Frameshift
			*CXCR4*	c.1012C > T	p.(Arg338*)	45.1	Nonsense
			*EP300*	c.6316delA	p.(Met2106Cysfs*28)	11.1	Frameshift
			*EP300*	c.6329_6330insT	p.(Gln2110Hisfs*100)	9.9	Frameshift
			*MAL*	c.98T > G	p.(Phe33Cys)	8.7	Missense
			*KMT2D*	c.2886_2887delTGinsCA	p.(Ala963Thr)	13.6	Missense
			*MYC*	c.77_78delACinsGT	p.(Asn26Ser)	67.4	Missense
			*MYC*	c.63C > G	p.(Ser21Arg)	67.6	Missense
			*MYC*	c.214C > A	p.(Pro72Thr)	68.3	Missense
			*MYC*	c.175G > A	p.(Ala59Thr)	67.7	Missense
34	FFPE	FL	*BCL2*	c.256C > T	p.(Leu86Phe)	25.8	Missense
			*BCL2*	c.20C > A	p.(Thr7Lys)	20.2	Missense
			*BCL2*	c.185C > G	p.(Ser62Cys)	25	Missense
			*BCL2*	c.133G > A	p.(Ala45Thr)	20.8	Missense
			*BCL2*	c.469A > C	p.(Met157Leu)	25.2	Missense
			*EZH2*	c.1921T > A	p.(Tyr641Asn)	22.5	Missense
			*NOTCH2*	c.4609G > T	p.(Asp1537Tyr)	48.7	Missense
			*SOCS1*	c.416_418delinsCCG	p.(Gly139_ser140delinsAlaGly)	29.3	Missense
			*SOCS1*	c.348C > G	p.(Ser116Arg)	30.5	Missense
			*TNFRSF14*	c.3G > T	p.(Met1?)	22.4	No-start
			*TNFRSF14*	c.178 + 1G > T	p.(?)	10.5	Splice_donor_ + 1
			*TNFRSF14*	c.433_434dup	p.(Ser145Argfs*)	8.9	Frameshift
			*EP300*	c.4115G > A	p.(Cys1372Tyr)	10.7	Missense
			*FOXO1*	c.1A > T	p.(Met1?)	24.1	No-start
			*FOXO1*	c.358C > G	p.(Pro120Ala)	20.9	Missense
			*GNA13*	c.1A > T	p.(Met1?)	21.5	No-start
			*GNA13*	c.841C > G	p.(Leu281Val)	5	Missense
			*KMT2D*	c.15088C > T	p.(Arg5030Cys)	23.6	Missense
			*KMT2D*	c.8311C > T	p.(Arg2771*)	24.4	Nonsense
35	FFPE	DLBCL	*NRAS*	c.38G > T	p.(Gly13Val)	6.1	Missense
			*EZH2*	c.2060C > T	p.(Ala687Val)	35.4	Missense
			*REL*	c.392A > G	p.(Asn131Ser)	57.9	Missense
			*ARID1A*	c.2668A > G	p.(Met890Val)	31.8	Missense
			*ARID1A*	c.4540_4543delinsCCGT	p.(Thr1514_Gly1515delinsProCys)	9.7	Missense
			*EP300*	c.6329_6330insT	p.(Gln2110Hisfs*100)	22.2	Frameshift
			*EP300*	c.6323A > T	p.(Gln2108Leu)	22.7	Missense
			*EP300*	c.6316del	p.(Met2106Cysfs*28)	25.2	Frameshift
			*FOXO1*	c.62G > T	p.(Arg21Leu)	28.8	Missense
			*FOXO1*	c.118T > C	p.(Ser40Pro)	30.8	Missense
			*MAL*	c.98T > G	p.(Phe33Cys)	13.5	Missense
			*MEF2B*	c.32T > C	p.(Ile11Thr)	27.5	Missense
			*KMT2D*	c.6221_6224dupACAA	p.(Val2076Glnfs*7)	23.9	Frameshift
			*KMT2D*	c.12204_12207delACTC	p.(Ser4070Glyfs*25)	37.6	Frameshift
			*KMT2D*	c.2876delA	p.(Tyr959Serfs*41)	10.2	Frameshift
			*KMT2D*	c.10192A > G	p.(Met3398Val)	40.7	Missense
			*KMT2D*	c.2886_2887delTGinsCA	p.(Ala963Thr)	22.6	Missense
36	FFPE	FL	*EZH2*	c.1921T > A	p.(Tyr641Asn)	32.4	Missense
			*TNFRSF14*	c.42delC	p.(Thr15Profs*7)	29.5	Frameshift
			*ARID1A*	c.4899delC	p.(Met1634fs*1)	30.6	Frameshift
			*EP300*	c.631G > A	p.(Gly211Ser)	50.6	Missense
			*KMT2D*	c.5188_5782 + 1del	p.(?)	19.8	Splice_donor_ + 1
			*KMT2D*	c.15583C > T	p.(Gln5195*)	21	Nonsense
37	FFPE	DLBCL	*BCL6*	c.1760C > G	p.(Ala587Gly)	29.2	Missense
			*PLCG2*	c.2009T > G	p.(Leu670Arg)	7.1	Missense
			*POT1*	c.1315_1317del	p.(Ala439del)	6.4	Inframe
			*SOCS1*	c.195_206delGCGCATCACGCG	p.(Arg66_Arg69del)	34.7	Inframe
			*ARID1A*	c.4540_4543delACGGinsCCGT	p.(Thr1514_Gly1515delinsProCys)	16.6	Missense
			*CIITA*	c.2342_2345delCGGTinsTGGC	p.(Ser781_Val782delinsLeuAla)	45.5	Missense
			*EP300*	c.6329_6330insT	p.(Gln2110Hisfs*100)	18.2	Frameshift
			*EP300*	c.6316delA	p.(Met2106Cysfs*28)	18.7	Frameshift
			*EP300*	c.6323A > T	p.(Gln2108Leu)	19.1	Missense
			*FOXO1*	c.1478G > C	p.(Gly493Ala)	7.2	Missense
			*MAL*	c.98T > G	p.(Phe33Cys)	19.3	Missense
			*KMT2D*	c.13753_13757delinsTTGAC	p.(Val4585_Asn4586delinsLeuThr)	5.4	Missense
			*KMT2D*	c.2886_2887delTGinsCA	p.(Ala963Thr)	37.6	Missense
38	FFPE	DLBCL	*B2M*	c.2T > A	p.(Met1?)	31.9	No-start
			*NFKBIE*	c.1108 + 2T > A	p.(?)	25.1	Splice_donor_ + 2
			*NFKBIE*	c.759_762delTTAC	p.(Tyr254Serfs*13)	24.6	Frameshift
			*PRDM1*	c.1142A > G	p.(Tyr381Cys)	49.5	Missense
			*TNFRSF14*	c.632T > A	p.(Val211Asp)	32	Missense
			*CD58*	c.70 + 2T > G	p.(?)	34.2	Splice_donor_ + 2
39	FFPE	DLBCL	*BRAF*	c.1799T > A	p.(Val600Glu)	26.4	Missense
			*SOCS1*	c.49_52delGCAG	p.(Ala17Serfs*67)	26	Frameshift
			*CIITA*	c.2342_2345delCGGTinsTGGC	p.(Ser781_Val782delinsLeuAla)	45.6	Missense
			*KMT2A*	c.627G > T	p.(Lys209Asn)	24.8	Missense
			*KMT2D*	c.11180G > A	p.(Arg3727His)	50.7	Missense
40	FFPE	DLBCL	–	–	–	–	–
41	FFPE	FL	*MYD88*	c.909_929dup	p.(Ser304_Leu310dup)	7.1	Inframe
42			*SOCS1*	c.523C > T	p.(Gln175*)	8.9	Nonsense
			*ARID1A*	c.20_52del	p.(Ser11Leufs*89)	13.6	Frameshift
			*EP300*	c.631G > A	p.(Gly211Ser)	46	Missense
			*EP300*	c.3754A > G	p.(Arg1252Gly)	47.4	Missense
			*GNA13*	c.32T > C	p.(Leu11Pro)	24.2	Missense
			*MYC*	c.212_21dupTGC	p.(Leu71dup)	19.7	Inframe
43	FFPE	DLBCL	–				
44	FFPE	DLBCL	*TP53*	c.743G > A	p.(Arg248Gln)	12.6	Missense
			*TP53*	c.919 + 1G > A	p.(?)	12.6	Splice_donor_ + 1
			*PRDM1*	c.626_627del	p.(His209Leufs*25)	19.1	Frameshift
			*ARID1A*	c.60_62del	p.(Pro21del)	9.4	Inframe
			*MYD88*	c.719T > C	p.(Met240Thr)	35	Missense
45	FFPE	DLBCL	*TP53*	c.404G > T	p.(Cys135Phe)	7.2	Missense
			*KRAS*	c.38G > A	p.(Gly13Asp)	13.5	Missense
			*CARD11*	c.383C > T	p.(Thr128Met)	18.2	Missense
			*PIM1*	c.447G > T	p.(Trp149Cys)	15.9	Missense
			*PIM1*	c.451G > C	p.(Val151Leu)	16.4	Missense
			*PIM1*	c.242C > T	p.(Pro81Leu)	14.6	Missense
			*SOCS1*	c.430C > T	p.(Phe144Leu)	14.4	Missense
			*SOCS1*	c.534C > G	p.(Cys178Trp)	15.3	Missense
			*CCND3*	c.541_544dup	p.(Ser182*)	12.1	Nonsense
			*EP300*	c.865A > G	p.(Met289Val)	35.9	Missense
			*ID3*	c.203A > G	p.(Glu68Gly)	16.3	Missense
			*ID3*	c.243G > C	p.(Gln81His)	15.5	Missense
			*ID3*	c.305C > T	p.(Ala102Val)	17.2	Missense
46	FFPE	DLBCL	*TP53*	c.919 + 1G > T	p.(?)	22.7	Splice_donor_ + 1
			*TP53*	c.455_456delinsT	p.(Pro152Leufs*18)	11	Frameshift
			*TP53*	c.743G > A	p.(Arg248Gln)	8.3	Missense
			*PRDM1*	c.695G > A	p.(Ser232Asn)	20.8	Missense
			*SOCS1*	c.248_280del	p.(Pro83_Leu93del)	5.7	Inframe
			*SOCS1*	c.120_122delinsACG	p.(Pro41Arg)	8.4	Missense
			*SOCS1*	c.299C > T	p.(Thr100Ile)	7	Missense
			*SOCS1*	c.140C > T	p.(Ala47Val)	9.3	Missense
			*SOCS1*	c.347G > A	p.(Ser116Asn)	5.6	Missense
			*SOCS1*	c.233G > A	p.(Gly78Glu)	7.9	Missense
			*CD58*	c.66C > A	p.(Cys22*)	8.4	Nonsense
			*CIITA*	c.3344G > A	p.(Ser1115Asn)	15.2	Missense
			*EP300*	c.631G > A	p.(Gly211Ser)	6.4	Missense
47	FFPE	DLBCL	*PIM1*	c.72G > C	p.(Lys24Asn)	11.1	Missense
			*PIM1*	c.61C > T	p.(His21Tyr)	30.1	Missense
			*PIM1*	c.4C > G	p.(Leu2Val)	30.1	Missense
			*CCND3*	c.568dupC	p.(Arg190Profs*)	30.5	Frameshift
			*EP300*	c.631G > A	p.(Gly211Ser)	41.7	Missense
			*FOXO1*	c.290C > G	p.(Ala97Gly)	6.2	Missense
			*KMT2D*	c.14782C > A	p.(Pro4928Thr)	49.1	Missense
			*MYD88*	c.818T > C	p.(Leu273Pro)	32.8	Missense

In FL, 83% of the patients presented *BCL2* rearrangement and a total of 93 somatic alterations, with a median of 7.4 mutations per sample (range 2–22). The most frequently mutated genes were *KMT2D* (80%), *TNFRSF14* (48%), *CREBBP* (40%), *BCL2* (40%), *TNFAIP3* (32%), *SOCS1* (32%), *CARD11* (28%) and *EZH2* (28%) (Fig. 1). A total of (13/15) 87% FL samples presented mutations in epigenetic modifiers genes.

In contrast, 28% of the patients with DLBCL presented *BCL6* rearrangement, 25% presented c-*MYC* rearrangement, and 16% presented *BCL2* rearrangement, with 16% presenting double-hit lymphomas. Furthermore, the patients presented a total of 279 somatic variants with a median of 8.6 mutations (range 0–35). In the overall cohort (n = 32), the most frequently mutated genes were *SOCS1* (40%), *KMT2D* (40%), *EP300* (40%), and *c-MYC* (32%) (Fig. 1). Sixty-eight percent (22/32) of the patients presented mutations in epigenetic modifier genes.

When comparing the germinal center B-cell (GCB) DLBCLs (n = 17) with the activated B-cell (ABC) DLBCLs (n = 9), *PIM1* mutations were present only in the patients with GCB DLBCL (41% vs. 0%; *p* = 0.03), and *XPO1* mutations were present only in the patients with ABC DLBCL (22% vs. 0%; *p* = 0.08), with statistically significant differences (Supplementary Fig. 1).

When we analyzed the patients with high-grade DLBCLs-NOS (n = 5), those with double-hit/triple-hit (n = 4) DLBCLs, and those with DLBCL-NOS (n = 20), c*-MYC* and *TCF3* were more present in the high-grade DLBCLs-NOS than in the DLBCLs-NOS (44% vs. 15%, *p* = 0.1; and 22% vs. 0%, *p* = 0.089, respectively). Mutations in *EZH2* and *MAL* were more frequent in the high-grade double-hit DLBCLs (50% vs. 4%, *p* = 0.04; 75% vs. 12%, *p* = 0.02). Mutations in *TP53*, *TCF3*, and *CD58* were more frequent in the high-grade DLBCLs-NOS (60% vs. 20%, *p* = 0.11; 40% vs. 0%, *p* = 0.025; 40% vs. 8%, *p* = 0.12) (Supplementary Fig. 2).

When we compared the mutations in FL (n = 15) versus those in DLBCL (n = 32), we found that the variants in the following genes were more frequently present in FL than in DLBCL: *BCL2* (*p* = 0.003), *CREBBP* (*p* = 0.003), *KMT2D* (*p* = 0.012), and *TNFRS14* (*p* = 0.015), with significant differences. In contrast, *PIM1* variants (*p* = 0.033) were more frequent in DLBCLs (Fig. 1).

Recurrent mutations (1–3) were found in *ARID1A, B2M, BCL2, CIITA, CREBBP, EP300, EZH2, FOXO1, KMT2D, MAL, MYD88, NFKBIE, PIM1, SOCS1, STAT6, TP53,* and *XPO* (Table 1)*.* Only *EZH2* (p.Tyr641Asn/His/Phe), *CIITA* (p.Ser781_Val782delinsLeuAla), *EP300* (p.Gly211Ser), and *MAL* (p.Phe33Cys) presented more than 4 recurrent mutations (Supplementary Fig. 3).

The presence of more than 1 mutation in the same gene was detected in several genes including *MYC*, *SOCS1*, *PIM1*, *CIITA*, *KMT2D,* and *BCL2* (Table 1). Nine patients presented mutations in the c-*myc* protooncogene, 4 presented more than 1 mutation and concomitant with *MYC* translocation. Eighty-three percent (27/33) of the *MYC* mutations occurred in exon 2. The other genes with more than one variant were *SOCS1* (59 mutations in 17 patients), *PIM1* (28 mutations in 6 patients), *CIITA* (12 mutations in 8 patients), and *KMT2D* (36 mutations in 22 patients); all of these patients were diagnosed with DLBCL. Also, 4 patients with FL presented more than 2 mutations in *BCL2*, all with *BCL2* rearrangement.

### Mutational data in FFPE and cfDNA (n = 26)

**Figure 2:**
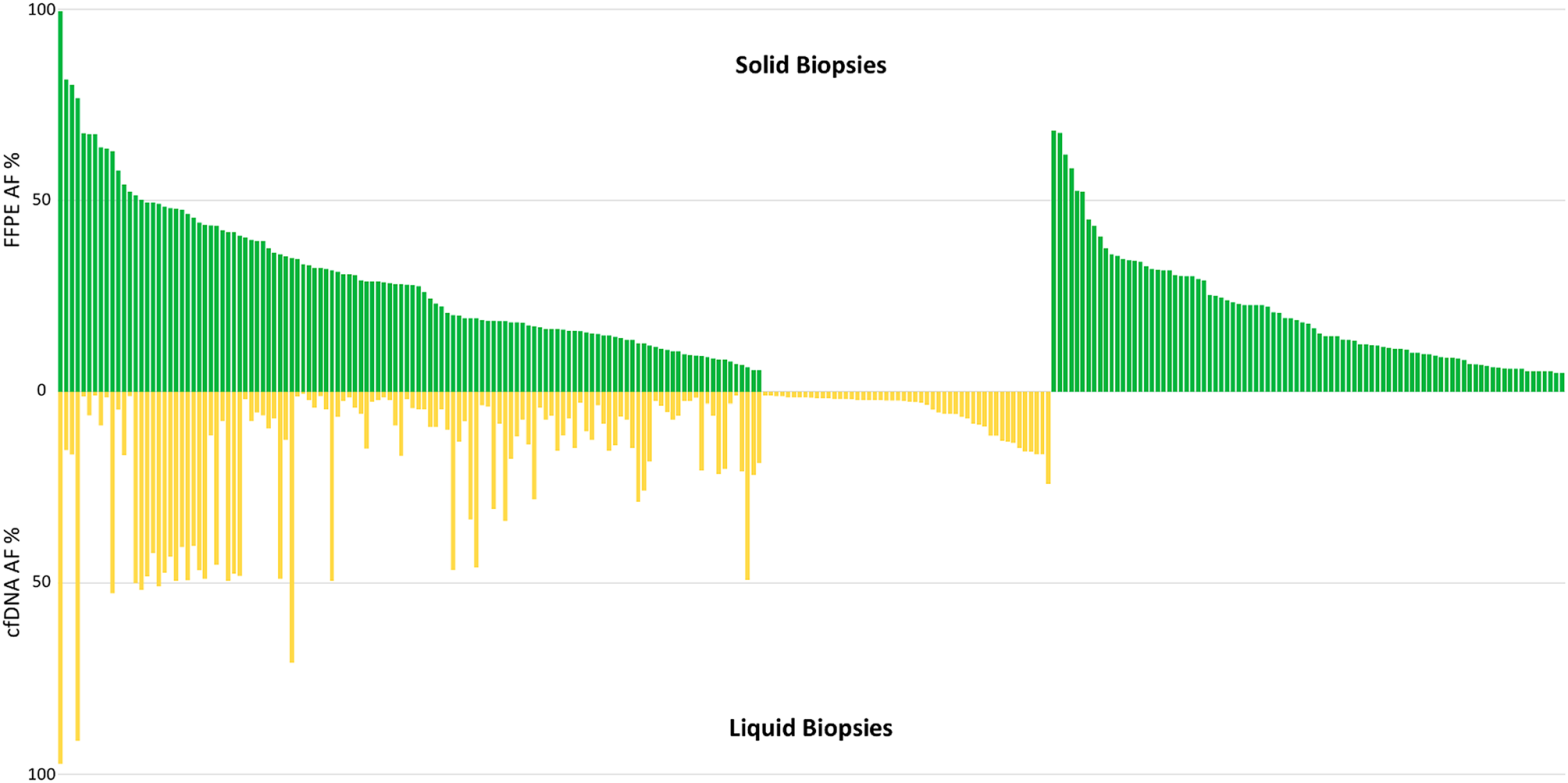
Concordance of mutations between solid and liquid biopsies. Allele frequencies (AF) are provided for the solid tissue biopsies (green bar plot) and for the liquid biopsies (yellow bar plot).

The cfDNA samples collected from the study patients at diagnosis were subjected to targeted sequencing (n = 26) (Table 2). In 92% (24/26) of the samples, we detected some variant in the free DNA in plasma. A total of 386 variants were detected (174 in the ctDNA samples and 212 in the FFPE samples). Of the total variants, 123 mutations (63.7%) were detected in both types of samples, 51 mutations were detected only in the ctDNA samples (13.2%), and 89 mutations were detected only in the FFPE samples (23%) (Fig. 2). Those variants that were detected in both types of samples had higher VAFs (28%) in the FFPE samples than in the ctDNA samples (17.9%). When considering only those mutations with VAFs > 10% in the FFPE samples, the percentage of mutations identified in both samples was 86%; specifically, the ctDNA samples that had a percentage of mutations < 50% had an input ctDNA concentration < 0.5 ng/µL (Supplementary Table 4). Overall, 96% (25/26) of the patients had at least one alteration observed in the ctDNA sample that was identical to that in the FFPE tissue sample.

**Table 2:** Mutational analysis of circulating tumor DNA and formalin-fixed paraffin-embedded tissue samples.

UPN	Diagnosis	Gene	c.DNA	Protein	FPPE VAF	cfDNA VAF	Consenquence
4	FL	*BCL2*	c.140G > A	p.(Gly47Asp)	28	2	Missense
		*BCL2*	c.175C > A	p.(Pro59Thr)	28.4	2.2	Missense
		*BCL2*	c.151T > G	p.(Ser51Ala)	28.8	2.6	Missense
		*EZH2*	c.1922A > T	p.(Tyr641Phe)	30.5	4	Missense
		*STAT6*	c.1256A > G	p.(Asp419Gly)	5.3	–	Missense
		*CD58*	c.254C > G	p.(Thr85Ser)	47.9	49.4	Missense
		*MEF2B*	c.170A > G	p.(Tyr57Cys)	16.8	4	Missense
		*KMT2D*	c.3931A > T	p.(Arg1311*)	42.2	7.6	Nonsense
		*KMT2D*	c.13893 + 2T > A	p.(?)	22.4	4.5	Splice_donor_ + 2
		*TNFRSF14*	c.139T > A	p.Tyr47Asn	–	1.1	Missense
9	DLBCL	*CDKN2A*	c.329G > A	p.(Trp110*)	6.9	–	Nonsense
		*KMT2D*	c.14450T > G	p.(Val4817Gly)	51.3	50	Missense
11	FL	*BCL2*	c.392C > G	p.(Ala131Gly)	11.7	2.5	Missense
		*BCL2*	c.517A > G	p.(Ile173Val)	9.4	1.6	Missense
		*CARD11*	c.748T > C	p.(Ser250Pro)	14.5	–	Missense
		*CREBBP*	c.4394A > G	p.(Tyr1465Cys)	32.1	4.5	Missense
		*CCND3*	c.531_532delCTinsTG	p.(Ser178Ala)	99.7	97.3	Missense
		*KMT2D*	c.6664C > T	p.(Gln2222*)	12.2	–	Nonsense
		*KMT2D*	c.5335A > T	p.(Lys1779*)	14.5	–	Nonsense
		*CXCR4*	c.1025C > G	p.(Ser342*)	–	1.5	Nonsense
		*KMT2A*	c.6664C > T	p.(Gln2222*)	–	1	Nonsense
13	DLBCL	*TP53*	c.490A > T	p.(Lys164*)	31.8	–	Nonsense
		*B2M*	c.2T > G	p.(Met1?)	52.4	–	No-start
		*PIM1*	c.676G > A	p.(Glu226Lys)	35.5	–	Missense
		*PIM1*	c.370C > T	p.(Pro124Ser)	62.1	–	Missense
		*PIM1*	c.434G > A	p.(Arg145His)	6.7	–	Missense
		*PIM1*	c.202C > T	p.(His68Tyr)	23.3	–	Missense
		*SOCS1*	c.8C > T	p.(Ala3Val)	30.3	–	Missense
		*FOXO1*	c.435del	p.(Ala146Argfs*187)	17.9	–	Frameshift
		*MEF2B*	c.78C > G	p.(Phe26Leu)	52.5	–	Missense
		*MYD88*	c.818T > C	p.(Leu273Pro)	33	2.1	Missense
14	FL	*BCL2*	c.191A > C	p.(Asp64Ala)	12	–	Missense
		*BCL2*	c.93T > C	p.(Asp31Glu)	9	–	Missense
		*TNFAIP3*	c.2014G > T	p.(Gly672*)	8.8	–	Nonsense
		*TNFRSF14*	c.463delA	p.(Thr155Profs*)	13.3	–	Frameshift
		*KMT2D*	c.172 + 2T > C	p.(?)	12.4	–	Splice_donor_ + 2
15	FL	*CREBBP*	c.4382T > C	p.(Leu1461Pro)	29.1	5.6	Missense
		*KMT2D*	c.16489_16491delATC	p.(Ile5479del)	32.4	4	Inframe
		*KMT2D*	c.9019delG	p.(Glu3007Lysfs*22)	26.1	4.5	Frameshift
18	DLBCL	*NFKBIE*	c.668_671delTGCTinsAGCG	p.(Leu223_Leu224delins*Arg)	20.6	9.8	Missense
		*SOCS1*	c.7G > A	p.(Ala3Thr)	14	6.4	Missense
		*SOCS1*	c.374G > C	p.(Ser125Thr)	19.1	7.7	Missense
		*SOCS1*	c.407A > C	p.(His136Pro)	18	7.2	Missense
		*SOCS1*	c.428G > A	p.(Ser143Asn)	13.5	7.2	Missense
		*SOCS1*	c.398delG	p.(Gly133Alafs*72)	10.6	7.2	Frameshift
		*SOCS1*	c.55C > T	p.(Pro19Ser)	14.6	8.3	Missense
		*SOCS1*	c.412G > C	p.(Asp138His)	15.9	7	Missense
		*SOCS1*	c.391C > G	p.(Gln131Glu)	16.4	7.3	Missense
		*SOCS1*	c.391C > T	p.(Gln131*)	16.4	6.3	Nonsense
		*SOCS1*	c.318C > G	p.(Ser106Arg)	8.7	6.2	Missense
		*SOCS1*	c.528G > C	p.(Glu176Asp)	10.6	6.3	Missense
		*ARID1A*	c.5012G > A	p.(Arg1671Gln)	18.4	8.3	Missense
		*MYC*	c.482C > T	p.(Ser161Leu)	10.9	5.2	Missense
		*MYC*	c.218_219delCCinsTA	p.(Thr73Ile)	11.2	3.6	Missense
		*MYC*	c.1164C > G	p.(Ser388Arg)	18.7	3.5	Missense
		*MYC*	c.557G > C	p.(Cys186Ser)	14.5	–	Missense
		*MYC*	c.895G > C	p.(Ala299Pro)	18.6	3.8	Missense
		*MYC*	c.910_999dup	p.(Lys304_Asp333dup)	40.6	–	Inframe
		*MYC*	c.654C > G	p.(Ser218Arg)	15.1	3.5	Missense
		*MYC*	c.785C > T	p.(Thr262Ile)	15.8	2.9	Missense
		*MYC*	c.68_71delinsGCAG	p.(Phe23Cys)	9.7	2.4	Missense
		*MYC*	c.63C > G	p.(Ser21Arg)	9.6	2.5	Missense
		*MYC*	c.162G > C	p.(Glu54Asp)	9	3.1	Missense
		*MYC*	c.144G > A	p.(Asp48Glu)	7.9	3.1	Missense
		*MYC*	c.358_361delinsTTGT	p.(Asp120Leu)	11.8	–	Missense
		*REL*	c.868A > G	p.(Lys290Glu)	–	5.7	Missense
		*MYC*	c.361C > T	p.(Asp121Tyr)	–	2.7	Missense
19	FL	*CARD11*	c.1202A > T	p.(Asp401Val)	19.9	13.1	Missense
		*SOCS1*	c.4G > T	p.(Val2Leu)	18.1	11.6	Missense
		*SOCS1*	c.14A > G	p.(Asn5Ser)	20	46.6	Missense
		*SOCS1*	c.134_139dupTCCCGG	p.(Val45_Pro46dup)	43.5	11.3	Inframe
		*TNFAIP3*	c.1035C > A	p.(Tyr345*)	28.9	14.8	Nonsense
		*TNFRSF14*	c.70G > T	p.(Val24Leu)	28.1	16.6	Missense
		*B2M*	c.1A > G	p.(Met1?)	–	6.9	No-start
		*B2M*	c.346 + 2T > A	p.(?)	–	1.5	Splice_donor_ + 2
		*B2M*	c.35T > C	p.(Leu12Pro)	–	3.3	Missense
		*CREBBP*	c.4406T > C	p.(Leu1469Pro)	–	1.9	Missense
		*KMT2D*	c.10867C > T	p.(Gln3623*)	–	2.3	Nonsense
		*MYD88*	c.719T > C	p.(Met240Thr)	–	4.7	Missense
21	DLBCL	*B2M*	c.176T > A	p.(Leu59*)	80.3	16.4	Nonsense
		*ATM*	c.8284C > T	p.(Gln2762*)	54.3	16.5	Nonsense
		*NFKBIE*	c.1147_1153delCAACCAC	p.(Gln383Serfs*46)	31.3	6.5	Frameshift
		*NFKBIE*	c.759_762delTTAC	p.(Tyr254Serfs*13)	33.4	.6	Frameshift
		*PRDM1*	c.75delG	p.(Arg25Serfs*13)	41.8	49.5	Frameshift
		*SOCS1*	c.358_361delGCCTinsCC	p.(Ala120Profs*?)	39.7	7.6	Frameshift
		*SOCS1*	c.434_437delACTG	p.(Asp145Alafs*59)	39.4	5.4	Frameshift
		*TNFAIP3*	c.2350C > T	p.(Gln784*)	64	8.7	Nonsense
		*TNFAIP3*	C.295 + 2T > C	p.(?)	67.5	6.1	Splice_donor_ + 2
		*CDKN2A*	c.394G > A	p.(Ala132Thr)	32.4	1.1	Missense
		*CIITA*	c.34_46delTACCTGTCAGAGC	p.(Tyr12Profs*15)	36.4	7	Frameshift
		*CIITA*	c.1652delG	p.(Gly551Alafs*7)	35.9	–	Frameshift
		*CIITA*	c.3262G > A	p.(Gly1880Arg)	5.3	–	Missense
		*FOXO1*	c.61C > T	p.(Arg21Cys)	39.4	6.1	Missense
		*GNA13*	c.179A > G	p.(Asp60Gly)	81.6	15.2	Missense
		*MYC*	c.25A > G	p.(Asn9Asp)	43.3	–	Missense
		*CIITA*	c.3317 + 2T > C	p.(?)	–	1.2	Splice_donor_ + 2
		*KMT2A*	c.137T > G	p.(Val46Gly)	–	1.4	Missense
22	FL	*BCL6*	c.1752C > A	p.(Asn584Lys)	27.8	4.2	Missense
		*CCND3*	c.613G > A	p.(Asp205Asn)	5.3	–	Missense
		*GNA13*	c.243_244del	p.(Glu82Glyfs*19)	5	–	Frameshift
		*MYC*	c.154_156del	p.(Lys51Del)	5.4	–	Inframe
23	DLBCL	*TP53*	c.839G > A	p.(Arg280Lys)	28.2	8.7	Missense
		*NFKBIE*	c.759_762delTTAC	p.(Tyr254Serfs*13)	12.3	–	Frameshift
		*CCND3*	c.626T > C	p.(Ile209Thr)	23.1	9.1	Missense
		*CCND3*	c.604A > C	p.(Thr202Pro)	24.4	9.1	Missense
24	DLBCL	*BCL2*	c.17G > A	p.(Arg6Lys)	5.9	–	Missense
		*B2M*	c.16G > C	p.(Ala6Pro)	49.4	42.2	Missense
		*TNFRSF14*	c.49_50delinsCAG	p.(Lys17Glnfs*60)	8.9	–	Frameshift
		*CXCR4*	c.1025C > A	p.(Ser342*)	5	–	Nonsense
		*MAL*	c.59C > T	p.(Thr20Ile)	46.6	49.3	Missense
		*B2M*	c.3G > C	p.(Met1?)	–	8.5	No-start
		*CREBBP*	c.4829_4830del	p.(Pro1610Hisfs*11)	–	2.1	Frameshift
		*NRAS*	c.38G > A	p.(Gly13Asp)	–	1	Missense
		*PAX5*	c.979T > C	p.(Tyr327His)	–	2.1	Missense
		*PIM1*	c.117G > T	p.(Gln39His)	–	5.4	Missense
26	DLBCL	*TP53*	c.725G > T	p.(Cys242Phe)	76.8	91.4	Missense
		*TCF3*	c.1688G > A	p.(Arg563His)	17.1	28.1	Missense
28	DLBCL	*CHD2*	c.1281G > A	p.(Trp427*)	18.6	30.6	Nonsense
		*MAL*	c.98T > G	p.(Phe33Cys)	6	–	Missense
		*MYC*	c.1148A > G	p.(Asn383Ser)	22.9	–	Missense
		*MYC*	c.490C > T	p.(Pro164Ser)	20.6	–	Missense
		*MYD88*	c.818T > C	p.(Leu273Pro)	18.4	33.8	Missense
		*MYD88*	c.797C > T	p.(Pro266Leu)	19.1	33.4	Missense
		*PIM1*	c.550C > T	p.(Leu184Phe)	–	5.7	Missense
		*PRDM1*	c.2182G > T	p.(Glu728*)	–	2.9	Nonsense
		*EP300*	c.3754A > G	p.(Arg1252Gly)	–	1.4	Missense
31	DLBCL	*BRAF*	c.1780G > A	p.(Asp594Asn)	29.4	–	Missense
		*EZH2*	c.1921T > A	p.(Tyr641Asn)	34.4	–	Missense
		*STAT6*	c.1255G > T	p.(Asp419Tyr)	52.4	1.1	Missense
		*PIM1*	c.409G > T	p.(Gly137*)	34	–	Nonsense
		*PIM1*	c.285G > C	p.(Lys95Asn)	31.8	–	Missense
		*SOCS1*	c.220C > G	p.(Leu74Val)	30.7	1.4	Missense
		*SOCS1*	c.178T > C	p.(Ser60Pro)	28.6	1.4	Missense
		*KMT2D*	c.14843C > G	p.(Ser4948*)	40.3	2	Nonsense
		*KMT2D*	c.7586delG	p.(Gly2529Alafs*14)	22.7	–	Frameshift
32	DLBCL	*NOTCH1*	c.7541_7542delCT	p.(Pro2514Argfs*4)	34.7	1.3	Frameshift
		*ARID1A*	c.4540_4543delACGGinsCCGT	p.(Thr1514_Gly1515delinsProCys)	10.1	–	Missense
		*MAL*	c.98T > C	p.(Phe33Cys)	6	–	Missense
		*KMT2D*	c.2886_2887delTGinsCA	p.(Ala963Thr)	11.5	–	Missense
		*MEF2B*	c.928T > G	p.(Ser310Ala)	–	2	Missense
33	DLBCL	*NFKBIE*	c.98C > T	p.(Ser33Phe)	63	52.6	Missense
		*CCND3*	c.544_554dupTCCAGCCCAGC	p.(Lys187Alafs*?)	63.7	1.5	Frameshift
		*CXCR4*	c.1012C > T	p.(Arg338*)	45.1	–	Nonsense
		*EP300*	c.6316delA	p.(Met2106Cysfs*28)	11.1	–	Frameshift
		*EP300*	c.6329_6330insT	p.(Gln2110Hisfs*100)	9.9	–	Frameshift
		*MAL*	c.98T > G	p.(Phe33Cys)	8.7	–	Missense
		*KMT2D*	c.2886_2887delTGinsCA	p.(Ala963Thr)	13.6	–	Missense
		*MYC*	c.77_78delACinsGT	p.(Asn26Ser)	67.4	1	Missense
		*MYC*	c.63C > G	p.(Ser21Arg)	67.6	1.3	Missense
		*MYC*	c.214C > A	p.(Pro72Thr)	68.3	–	Missense
		*MYC*	c.175G > A	p.(Ala59Thr)	67.7	–	Missense
35	DLBCL	*NRAS*	c.38G > T	p.(Gly13Val)	6.1	–	Missense
		*EZH2*	c.2060C > T	p.(Ala687Val)	35.4	12.5	Missense
		*REL*	c.392A > G	p.(Asn131Ser)	57.9	4.6	Missense
		*ARID1A*	c.2668A > G	p.(Met890Val)	31.8	49.4	Missense
		*ARID1A*	c.4540_4543delinsCCGT	p.(Thr1514_Gly1515delinsProCys)	9.7	–	Missense
		*EP300*	c.6329_6330insT	p.(Gln2110Hisfs*100)	22.2	–	Frameshift
		*EP300*	c.6323A > T	p.(Gln2108Leu)	22.7	–	Missense
		*EP300*	c.6316del	p.(Met2106Cysfs*28)	25.2	–	Frameshift
		*FOXO1*	c.62G > T	p.(Arg21Leu)	28.8	2.2	Missense
		*FOXO1*	c.118T > C	p.(Ser40Pro)	30.8	2.5	Missense
		*MAL*	c.98T > G	p.(Phe33Cys)	13.5	–	Missense
		*MEF2B*	c.32T > C	p.(Ile11Thr)	27.5	4.5	Missense
		*KMT2D*	c.6221_6224dupACAA	p.(Val2076Glnfs*7)	23.9	–	Frameshift
		*KMT2D*	c.12204_12207delACTC	p.(Ser4070Glyfs*25)	37.6	9.6	Frameshift
		*KMT2D*	c.2876delA	p.(Tyr959Serfs*41)	10.2	–	Frameshift
		*KMT2D*	c.2886_2887delTGinsCA	p.(Ala963Thr)	22.6	–	Missense
		*KMT2D*	c.4279T > G	p.(Cys1427Gly)	–	2.6	Missense
37	DLBCL	*BCL6*	c.1760C > G	p.(Ala587Gly)	29.2	–	Missense
		*PLCG2*	c.2009T > G	p.(Leu670Arg)	7.1	–	Missense
		*POT1*	c.1315_1317del	p.(Ala439del)	6.4	–	Inframe
		*SOCS1*	c.195_206delGCGCATCACGCG	p.(Arg66_Arg69del)	34.7	–	Inframe
		*ARID1A*	c.4540_4543delACGGinsCCGT	p.(Thr1514_Gly1515delinsProCys)	16.6	–	Missense
		*EP300*	c.6329_6330insT	p.(Gln2110Hisfs*100)	18.2	–	Frameshift
		*EP300*	c.6316delA	p.(Met2106Cysfs*28)	18.7	–	Frameshift
		*EP300*	c.6323A > T	p.(Gln2108Leu)	19.1	–	Missense
		*FOXO1*	c.1478G > C	p.(Gly493Ala)	7.2	–	Missense
		*MAL*	c.98T > G	p.(Phe33Cys)	19.3	–	Missense
		*KMT2D*	c.13753_13757delinsTTGAC	p.(Val4585_Asn4586delinsLeuThr)	5.4	–	Missense
		*KMT2D*	c.2886_2887delTGinsCA	p.(Ala963Thr)	37.6	–	Missense
38	DLBCL	*B2M*	c.2T > A	p.(Met1?)	31.9	–	No-start
		*NFKBIE*	c.1108 + 2T > A	p.(?)	25.1	–	Splice_donor_ + 2
		*NFKBIE*	c.759_762delTTAC	p.(Tyr254Serfs*13)	24.6	–	Frameshift
		*PRDM1*	c.1142A > G	p.(Tyr381Cys)	49.5	48.3	Missense
		*TNFRSF14*	c.632T > A	p.(Val211Asp)	32	–	Missense
		*CD58*	c.70 + 2T > G	p.(?)	34.2	–	Splice_donor_ + 2
40	DLBCL	–	–	–	–	–	–
43	DLBCL	*TNFAIP3*	c.1939A > C	p.(Thr647Pro)	50.2	51.8	Missense
		*SOCS1*	c.529C > G	p.(Leu177Val)	–	2.1	Missense
		*SOCS1*	c.174C > G	p.(Phe58Leu)	–	2.3	Missense
		*SOCS1*	c.598C > G	p.(Leu200Val)	–	1.8	Missense
		*SOCS1*	c.614G > C	p.(Ser205Thr)	–	1.9	Missense
		*SOCS1*	c.46_49delinsTCAA	p.(Ala16_Ala17delinsSerThr)	–	2.4	Missense
		*SOCS1*	c.22G > C	p.(Ala8Pro)	–	2.2	Missense
		*SOCS1*	c.4G > C	p.(Val2Leu)	–	2.2	Missense
		*MEF2B*	c.928T > G	p.(Ser310Ala)	–	1.7	Missense
44	DLBCL	*TP53*	c.743G > A	p.(Arg248Gln)	12.6	28.7	Missense
		*TP53*	c.919 + 1G > A	p.(?)	12.6	25.8	Splice_donor_ + 1
		*PRDM1*	c.626_627del	p.(His209Leufs*25)	19.1	46	Frameshift
		*ARID1A*	c.60_62del	p.(Pro21del)	9.4	–	Inframe
		*MYD88*	c.719T > C	p.(Met240Thr)	35	70.9	Missense
45	DLBCL	*TP53*	c.404G > T	p.(Cys135Phe)	7.2	1	Missense
		*KRAS*	c.38G > A	p.(Gly13Asp)	13.5	14.7	Missense
		*CARD11*	c.383C > T	p.(Thr128Met)	18.2	17.5	Missense
		*PIM1*	c.447G > T	p.(Trp149Cys)	15.9	14.7	Missense
		*PIM1*	c.451G > C	p.(Val151Leu)	16.4	15.3	Missense
		*PIM1*	c.242C > T	p.(Pro81Leu)	14.6	15.3	Missense
		*SOCS1*	c.430C > T	p.(Phe144Leu)	14.4	13.9	Missense
		*SOCS1*	c.534C > G	p.(Cys178Trp)	15.3	12.4	Missense
		*CCND3*	c.541_544dup	p.(Ser182*)	12.1	18.1	Nonsense
		*EP300*	c.865A > G	p.(Met289Val)	35.9	48.9	Missense
		*ID3*	c.203A > G	p.(Glu68Gly)	16.3	11.3	Missense
		*ID3*	c.243G > C	p.(Gln81His)	15.5	10.3	Missense
		*ID3*	c.305C > T	p.(Ala102Val)	17.2	13.7	Missense
		*TP53*	c.800G > T	p.(Arg267Leu)	–	2.3	Missense
		*MEF2B*	c.928T > G	p.(Ser310Ala)	–	1.6	Missense
46	DLBCL	*TP53*	c.919 + 1G > T	p.(?)	22.7	–	Splice_donor_ + 1
		*TP53*	c.455_456delinsT	p.(Pro152Leufs*18)	11	–	Frameshift
		*TP53*	c.743G > A	p.(Arg248Gln)	8.3	–	Missense
		*PRDM1*	c.695G > A	p.(Ser232Asn)	20.8	–	Missense
		*SOCS1*	c.248_280del	p.(Pro83_Leu93del)	5.7	21.8	Inframe
		*SOCS1*	c.120_122delinsACG	p.(Pro41Arg)	8.4	21.4	Missense
		*SOCS1*	c.299C > T	p.(Thr100Ile)	7	20.7	Missense
		*SOCS1*	c.140C > T	p.(Ala47Val)	9.3	20.6	Missense
		*SOCS1*	c.347G > A	p.(Ser116Asn)	5.6	18.5	Missense
		*SOCS1*	c.233G > A	p.(Gly78Glu)	7.9	25.2	Missense
		*CD58*	c.66C > A	p.(Cys22*)	8.4	20.1	Nonsense
		*CIITA*	c.3344G > A	p.(Ser1115Asn)	15.2	–	Missense
		*EP300*	c.631G > A	p.(Gly211Ser)	6.4	49.2	Missense
		*TP53*	c.845G > A	p.(Arg282Gln)	–	24	Missense
		*SOCS1*	c.522_523delinsAT	p.(Gln175*)	–	16.3	Nonsense
		*SOCS1*	c.435_*98del	p.(Asp145_*212del)	–	1.9	Frameshift
		*SOCS1*	c.385_388delinsTATA	p.(His129Phe130insTyrIle)	–	8.3	Missense
		*SOCS1*	c.466_469delinsACGC	p.(Ala156_Ala157delinsThrPro)	–	14.6	Missense
		*SOCS1*	c.37G > C	p.(Val13Leu)	–	12.7	Missense
		*SOCS1*	c.363_364delinsAC	p.(Gly122Arg)	–	16.2	Missense
		*SOCS1*	c.528_531delinsCCTA	p.(Gly176Asp)	–	15.7	Missense
		*SOCS1*	c.454G > A	p.(Glu152Lys)	–	13	Missense
		*SOCS1*	c.574G > A	p.(Ala192Thr)	–	11.4	Missense
		*SOCS1*	c.374G > A	p.(Ser125Asn)	–	11.5	Missense
		*SOCS1*	c.484C > A	p.(Ser162Met)	–	15.5	Missense
		*SOCS1*	c.46_49delinsACAC	p.(Ala16_Ala17delinsThrPro)	–	13.4	Missense
		*SOCS1*	c.429C > G	p.(Ser143Arg)	–	9	Missense
		*SOCS1*	c.614_617delinsATTA	p.(Ser205_206delinsAsnTyr)	–	6.5	Missense
		*SOCS1*	c.541C > T	p.(Arg181Cys)	–	1.7	Missense
		*SOCS1*	c.622C > T	p.(Pro208Ser)	–	5.8	Missense
47	DLBCL	*PIM1*	c.72G > C	p.(Lys24Asn)	11.1	–	Missense
		*PIM1*	c.61C > T	p.(His21Tyr)	30.1	–	Missense
		*PIM1*	c.4C > G	p.(Leu2Val)	30.1	–	Missense
		*CCND3*	c.568dupC	p.(Arg190Profs*)	30.5	–	Frameshift
		*FOXO1*	c.290C > G	p.(Ala97Gly)	6.2	–	Missense
		*KMT2D*	c.14782C > A	p.(Pro4928Thr)	49.1	50.9	Missense
		*MYD88*	c.818T > C	p.(Leu273Pro)	32.8	–	Missense

We found that the median number of mutations detected in ctDNA was higher among the stage III and IV patients than the early-stage patients (6 vs. 2.5 mutations, *p* = 0.05) and in the patients with bulky disease (7 vs. 3 mutations, *p* = 0.04) (Supplementary Fig. 4).

For the 51 variants detected only in ctDNA (12 patients, 9 DLBCL and 3 FL), the median VAF was lower than those that were also identified in the FFPE samples (2.5 vs. 9.1%). Interestingly, there were 5 patients harboring more than 2 mutations in the ctDNA samples that were not detected in their matched FFPE samples (UPN of 19, 24, 28, 43, 46). Four of these patients presented bulky disease and were stage III at diagnosis.

The mean baseline ctDNA concentration was 42.803 hGE/mL (range 0–635.152) at diagnosis (Supplementary Table 5). Higher ctDNA levels were also correlated with bulky disease (4.369 vs. 15.852 hGE/mL, *p* = 0.016). There were no differences based on the stage (Supplementary Fig. 4).

## Discussion

The optimal assessment of NHL includes morphological and immunophenotypic studies and chromosome and molecular analyses. NGS techniques provide relevant additional data for diagnosis, prognosis, and therapeutic management. Although NGS data on lymphomas require further validation before being implemented in daily practice, their clinical application is just around the corner. Numerous studies over the past decade have analyzed hundreds of tumor genomes of DLBCLs and FLs to better understand the molecular pathogenesis of these diseases^3–5,11–13^. In this study, we validated an NGS panel for DLBCL and FL in FFPE and ctDNA samples at diagnosis.

As one might expect of a cancer derived from cells and an environment of combinatorial diversity, heterogeneity is a defining characteristic of FL and DLBCL. We detected 372 pathogenic variants in 54 genes in 47 of the FFPE samples (93 in FL [median of 7.4 variants] and 279 in DLBCL [median of 8.6 variants]). In our study, 83% of the patients with FL presented BCL2 rearrangements, and the variants most frequently detected were *KMT2D*, *TNFRSF14*, *CREBBP*, *BCL2*, *TNFAIP3*, *SOCS1*, *CARD11,* and *EZH2*. Eighty-seven percent FL samples presented mutations in epigenetic modifier genes. These results agree with those from previous studies in the literature^14–16^. Twenty-eight percent of the patients with DLBCL presented *BCL6* rearrangement, 25% presented c-*MYC* rearrangement, and 16% presented *BCL2* rearrangement, with 16% presenting double-hit lymphomas. However, c-MYC rearrangement might be over-represented in our cohort compared with that described in the literature, given that the cases were not selected consecutively^17,18^. The variants most frequently detected were present in *SOCS1, KMT2D, EP300, c-MYC* and *TP53*, and 68% of the samples presented mutations in epigenetic modifier genes.

The mutational profile of DLBCL differs depending on the cell of origin. While GCB DLBCL is characterized by frequent translocations of *BCL2* and mutations of the epigenetic modifiers *CREBBP* and *EZH2*, these abnormalities are rare in ABC DLBCL. In contrast, mutations in genes encoding proteins implicated in B-cell receptor signaling and the nuclear factor kappa-light-chain-enhancer of activated B cells pathway (such as *CD79b* and *MYD88*) and genes involved in the regulation of the cell cycle (such as *CDKN2A*) contribute to the molecular pathogenesis of ABC DLBCL^5,19,20^. Our study found differentiated genetic profiles according to the GCB and ABC subtype. *BCL2* rearrangement, *EZH2*, *PIM1*, *CD58*, and *NFKBIE* were present only in the GCB subtype while *XPO1* was present only in ABC. Also, different profiles were observed in those patients classified as having high-grade lymphomas, where mutations in *EZH2* and *MAL* were more frequent in high-grade double-hit lymphomas and mutations in *TP53, TCF3* and *CD58* in high-grade NOS lymphomas. More extensive and complex panels than the ones used in this study are needed to adequately perform the molecular classification^4,5^. However, it is not entirely clear which strategy will be the most appropriate for clinical practice: large panels of genes, exomes, or whole genomes. What is clear is that, by including genetic analyses of lymphomas, we will be able to reach a much more certain diagnosis by establishing genetic risk profiles, as is the case for other hematological neoplasms such as acute leukemia, thus bringing us closer to more personalized care.

Undoubtedly, the paradigm of lymphoma diagnosis has changed since the incorporation of ctDNA. In addition to the genetic studies already performed on solid biopsies, we have the option of performing these genetic studies on non-invasive samples such as liquid biopsies. This type of sample has been increasingly used for a variety of applications in oncology, including diagnosis, prognosis, and the identification of therapeutic targets^10^. In addition, ctDNA provides information on tumor burden and the dynamics of treatment response^21,22^. Our study assessed the utility of liquid biopsy in B-cell lymphomas in routine clinical practice through the validation of a commercial gene panel in patients with lymphoma at diagnosis. Including 26 patients, we showed that the use of liquid biopsies is feasible in routine clinical practice for DLBCL and FL. Specifically, ctDNA was detectable in 92% of the patients, and in 96% of the cases we were able to identify at least 1 alteration in ctDNA that was identical to the FFPE at diagnosis, indicating the potentially universal applicability of ctDNA. When explaining the reasons for the differences found between FFPE and plasma samples, we believe that they have to do mainly with the quality of the sample and the characteristics of the tumor. In our study, some mutations present in FFPE were not detected in plasma samples, probably due to a low total amount of plasma used (< 5 ml) and, therefore, the quantity of ctDNA obtained was insufficient in a few cases. It is also true that localized diseases or those with a low tumor burden could release a small amount of ctDNA into plasma, so we have learned that a volume of at least 10 ml of plasma should be used for optimal analysis. On the contrary, mutations detected in plasma and not in FFPE may be due to the heterogeneity of the tumor, taking into account that we are analyzing only a small fragment of tissue and not the entire tumor, so not all clones would be represented. Different is with the liquid biopsy, where from all the existing lesions DNA is being released into the bloodstream.

Although various studies have shown the usefulness of these techniques in specialized centers^8,23^, particularly in clinical trials, the applicability of this technique in routine clinical practice has rarely been reported. Numerous reviews on the subject have listed the potential benefits of liquid biopsy^20,23,24^, both in the diagnosis and follow-up of NHL; however, the standardization of these tools is not yet a reality.

As previously described, we found a correlation between advanced stage and bulky disease and the number of ctDNA mutations^23,25^. Our analysis also found mutations in the liquid biopsy from patients at localized stages and with low tumor burden, which means that this tool can also be used in this patient group. As previously mentioned, not less than 10 ml must be used, in order to obtain a greater amount of DNA and thus be able to identify all mutations. Moreover, we found that patients with bulky disease had more mutations found only in ctDNA (i.e., not in the FFPE samples), which could indicate that ctDNA samples better represent the tumor’s genetic variability than standard biopsies. The possibility of finding a different mutational profile when comparing liquid biopsies and FFPE samples from the same patient has already been demonstrated by Sherer et al.^8^, who identified transformed FL in a liquid biopsy sample from a patient with low-grade FL, which had not been previously identified in the paraffin biopsy. Liquid biopsy could therefore be a useful strategy when looking for specific mutations for target molecules, especially in patients with bulky disease.

In conclusion, our results confirm that the NGS techniques provides additional relevant data at the time of diagnosis, not only in FFPE samples but also in ctDNA, both complementary, and also the liquid biopsy provides the extra of how easy it is to obtain. These ctDNA samples are useful not only in patients with advanced stages and large masses, but also provide information in patients with localized disease and low tumor burden. Although there is still a lack of standardization today, it is important that we begin to incorporate these techniques into clinical practice, given the valuable information they can offer us about the lymphoma.

## Supplementary Information

Supplementary Figure 1.

Supplementary Figure 2.

Supplementary Figure 3.

Supplementary Figure 4.

Supplementary Figure 5.

Supplementary Table 1.

Supplementary Table 2.

Supplementary Table 3.

Supplementary Table 4.

Supplementary Table 5.

Supplementary Legends.
