# Supplementary figures and images for: Incorporation of next-generation sequencing in clinical practice using solid and liquid biopsy for patients with non-Hodgkin’s lymphoma
Source: Sci Rep. 2021 Nov 24;11:22815. doi: 10.1038/s41598-021-02362-4 (PMC8613247; doi:10.1038/s41598-021-02362-4)

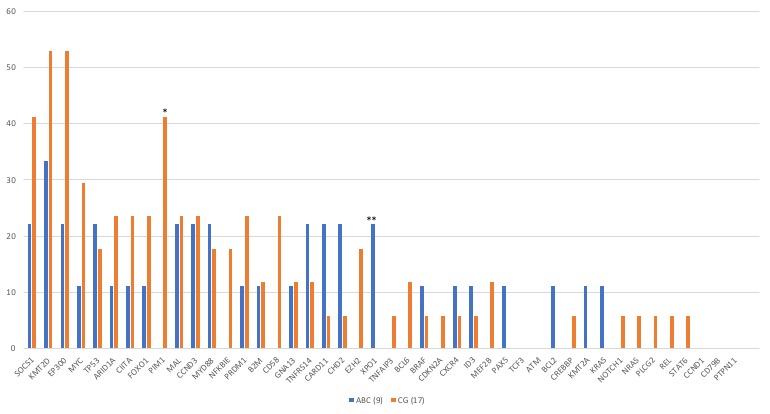

Supplement: Supplementary file 1 — Supplementary Figure 1. [file 41598_2021_2362_MOESM1_ESM.jpg]

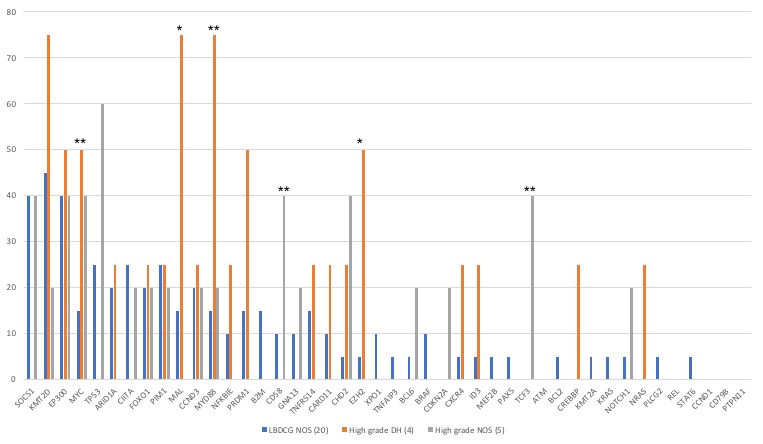

Supplement: Supplementary file 2 — Supplementary Figure 2. [file 41598_2021_2362_MOESM2_ESM.jpg]

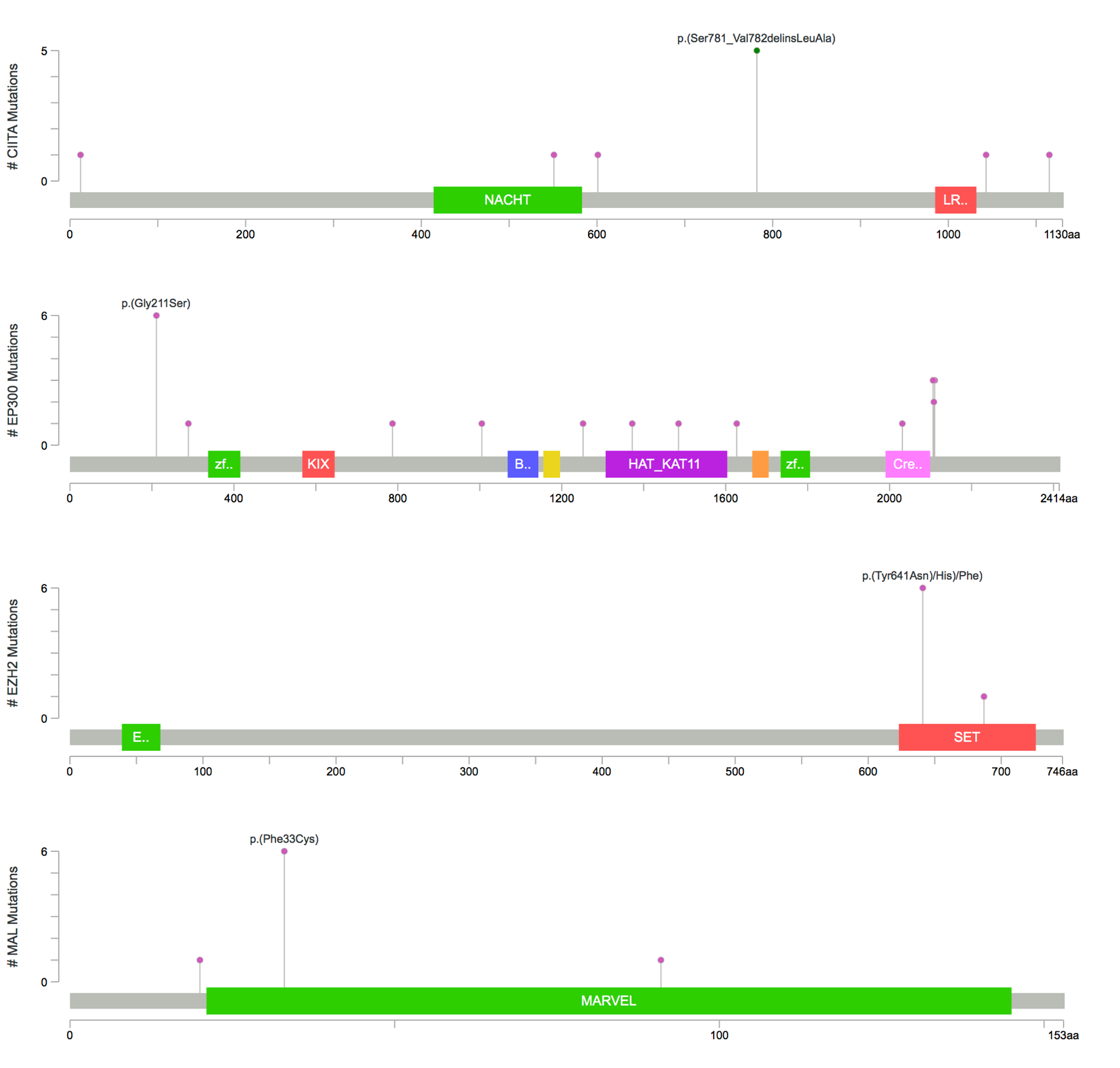

Supplement: Supplementary file 3 — Supplementary Figure 3. [file 41598_2021_2362_MOESM3_ESM.png]

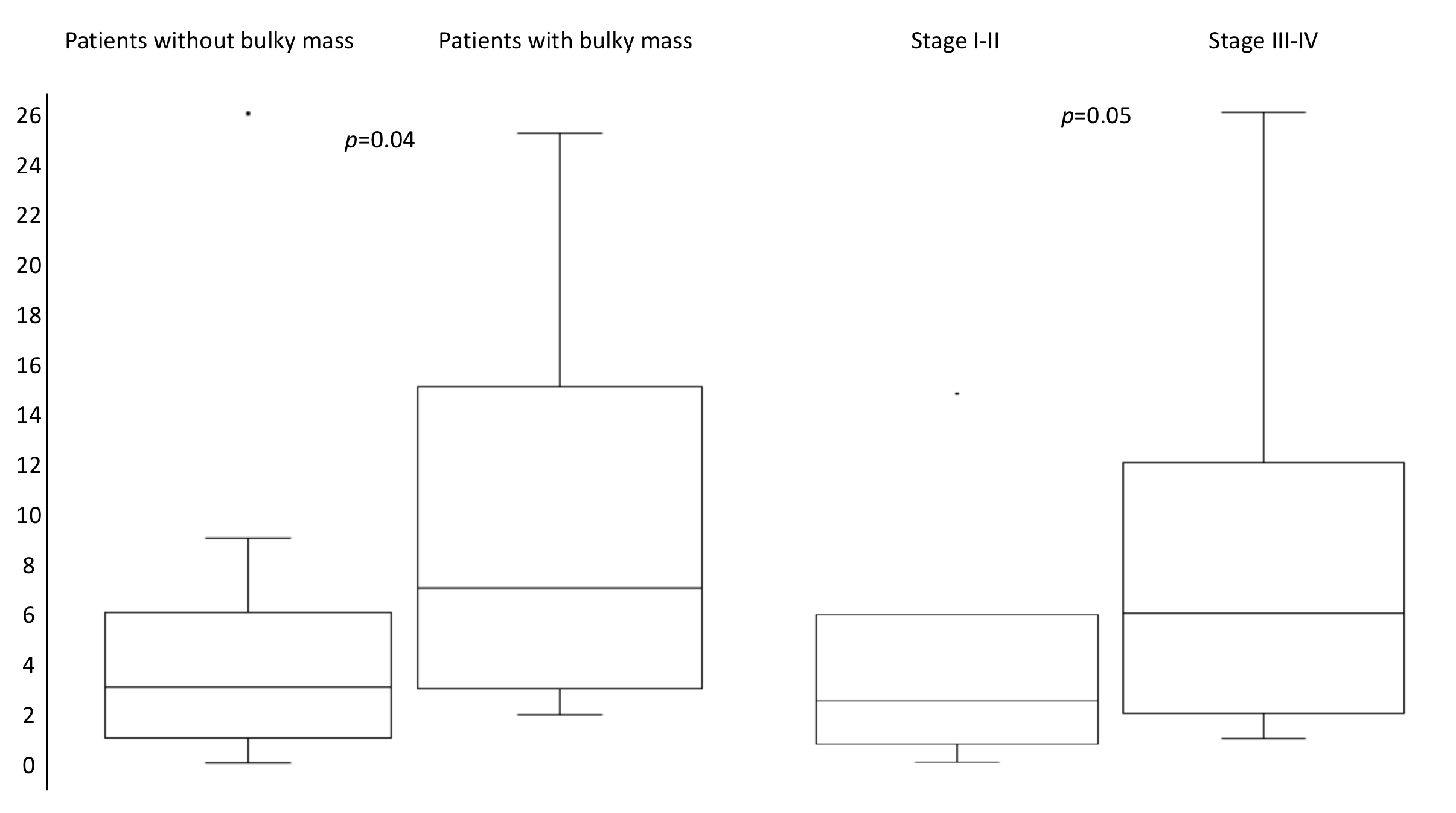

Supplement: Supplementary file 4 — Supplementary Figure 4. [file 41598_2021_2362_MOESM4_ESM.png]

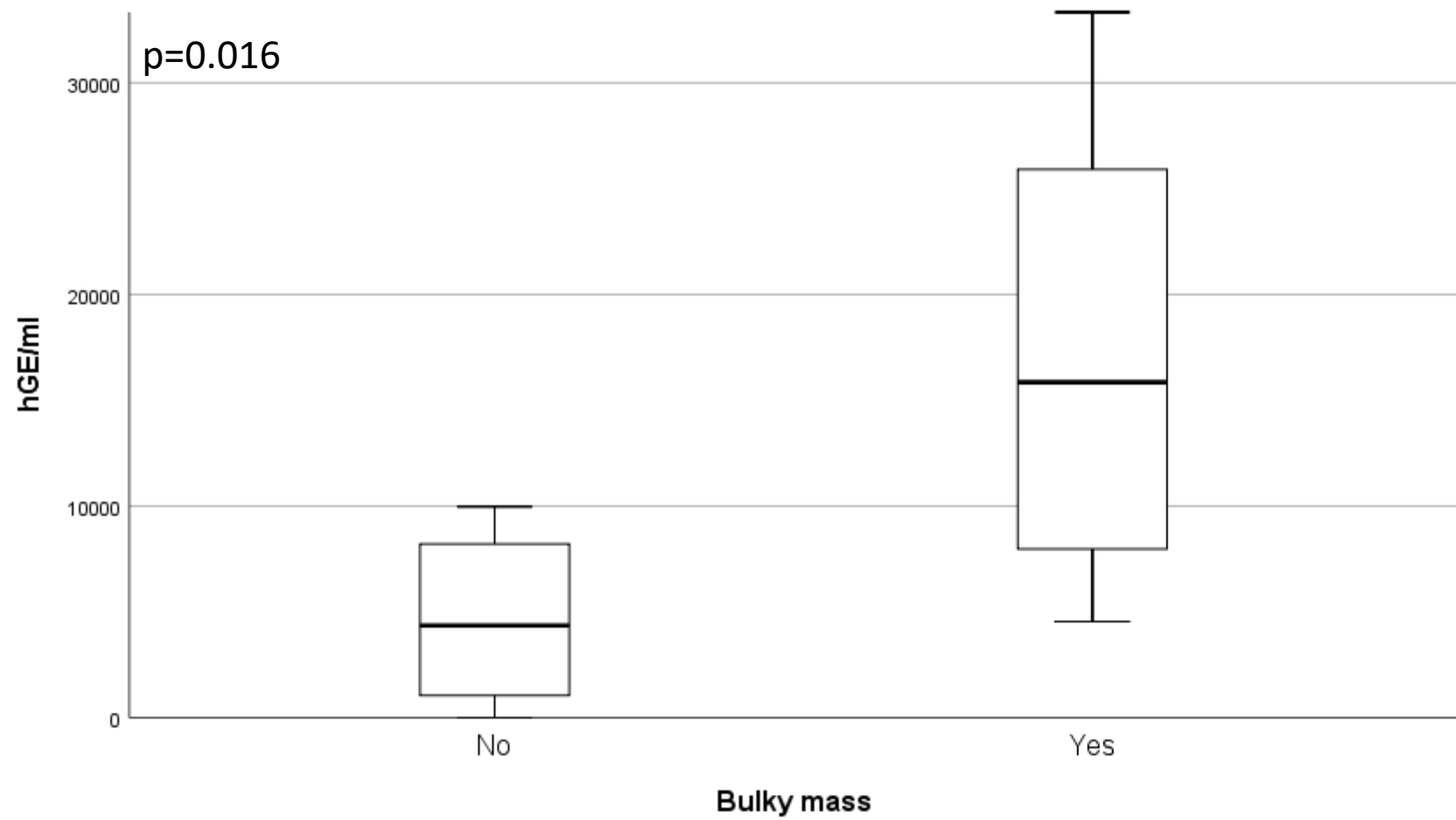

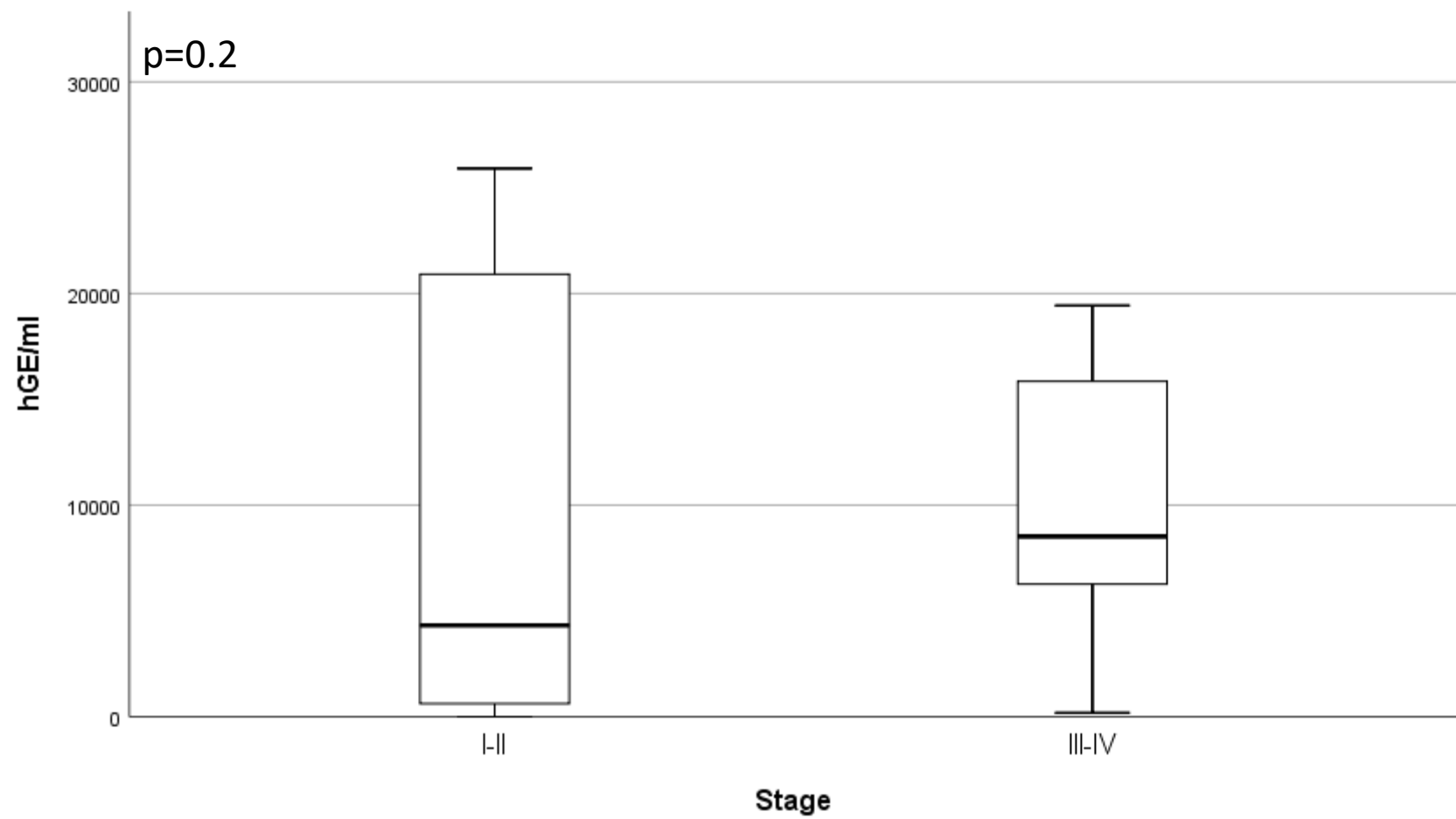

Supplement: Supplementary file 5 — Supplementary Figure 5. [file 41598_2021_2362_MOESM5_ESM.pdf]
