# Supplementary material for: Incorporation of next-generation sequencing in clinical practice using solid and liquid biopsy for patients with non-Hodgkin’s lymphoma
Source: Sci Rep. 2021 Nov 24;11:22815. doi: 10.1038/s41598-021-02362-4 (PMC8613247; doi:10.1038/s41598-021-02362-4)
Supplement: Supplementary file 11 — Supplementary Legends. [file 41598_2021_2362_MOESM11_ESM.docx]

**Supplementary information**

Supplementary Tables

Supplementary Table 1. Clinical characteristics of the patients with diffuse large B-cell lymphoma (n=32)

Supplementary Table 2. Clinical characteristics of the patients with follicular lymphoma (n=15)

Supplementary Table 3. Gene list

Supplementary Table 4. Gene panel features

Supplementary Table 5. Calculations of circulating tumor DNA concentrations, expressed in haploid genome equivalents (hGE) per mL of plasma (hGE/mL).

Supplementary Figures

Supplementary Figure 1. Frequencies of mutated genes in the diffuse large B-cell lymphoma - not otherwise specified (n=18) and high-grade diffuse large B-cell lymphoma (n=9). Statistically differences between follicular lymphoma and diffuse large B-cell lymphoma (p<0.05*) (p<0.1**).

Supplementary Figure 2. Frequencies of mutated genes in the germinal center diffuse large B-cell lymphoma (n=17) and activated diffuse large B-cell lymphoma (n=9). Statistically differences between follicular lymphoma and diffuse large B-cell lymphoma (p<0.05*) (p<0.1**).

Supplementary Figure 3. Recurrent mutations at the protein level. “Lollipop” diagrams display the localization and frequency of mutations in genes with recurrent mutations (over 4 mutations).

Supplementary Figure 4. Box-plot graph of the number of mutations detected in liquid biopsy depending on the stage of the disease and the presence of bulky mass.

Supplementary Figure 5. Box-plot graph of the concentration of ctDNA depending on the disease stage and the presence of bulky mass.
